# Estimating Individualized Survival Benefit of Consolidative Metastasis‐Directed Radiotherapy Following Chemoimmunotherapy in Nasopharyngeal Carcinoma With Exclusive Distant Metastasis

**DOI:** 10.1002/mco2.71089

**Published:** 2026-10-23

**Authors:** Xuguang Wang, Shuwei Chen, Xing Zhang, Linfei Mao, Jingtao Chen, Shida Yan, Jiawei Xie, Xianlu Gao, Liji Zheng, Yujie Zeng, Shiting Zhang, Zekun Deng, Mingyuan Du, Ming Song

**Affiliations:** ^1^ Department of Head and Neck Surgery Sun Yat‐sen University Cancer Center Guangzhou China; ^2^ State Key Laboratory of Oncology in South China Guangzhou China; ^3^ Guangdong Provincial Clinical Research Center for Cancer Guangzhou China; ^4^ Department of Neurosurgery National Cancer Center/National Clinical Research Center for Cancer/Cancer Hospital Chinese Academy of Medical Sciences and Peking Union Medical College Beijing China

**Keywords:** causal machine learning, clinical decision support system, metastasis‐directed radiotherapy, nasopharyngeal carcinoma

## Abstract

Optimal patient selection for consolidative metastasis‐directed radiotherapy (MDRT) after chemoimmunotherapy (CIT) remains uncertain in nasopharyngeal carcinoma (NPC) with exclusive distant metastasis, defined as distant metastasis without synchronous locoregional recurrence. This study was aimed at estimating the survival benefit of MDRT and identifying patients most likely to benefit. A total of 1083 patients from two independent centers were analyzed using a target trial emulation with cloning, artificial censoring, and inverse probability of censoring weighting. The emulation estimated a 4.2‐month gain in 3‐year progression‐free survival (PFS) restricted mean survival time with the MDRT strategy (95% CI: 2.4–5.9; bootstrap *p* < 0.001). Eight survival machine‐learning algorithms were developed and compared, among which the gradient boosting machine achieved 3‐year PFS AUROCs of 0.875 in internal validation and 0.877 in geographical external validation. Heterogeneous treatment effects were further estimated using a T‐learner framework across risk groups defined by recursive partitioning analysis. In the external cohort, model‐estimated 3‐year absolute risk reductions with MDRT were 21.6%, 14.7%, and 5.7% in intermediate‐, low‐, and high‐risk groups, respectively. The framework was subsequently implemented as a web‐based clinical decision‐support system. These findings support individualized selection of consolidative MDRT after CIT and provide a practical framework for multidisciplinary decision‐making.

## Graphical abstract

This study integrates target trial emulation and causal machine learning to individualize consolidative metastasis‐directed radiotherapy (MDRT) for exclusively distant metastatic nasopharyngeal carcinoma. Using a multicenter cohort of 1083 patients, we confirmed overall MDRT efficacy via a cloning–censoring–weighting approach. We then developed and compared multiple survival machine‐learning algorithms. A T‐learner framework with counterfactual risk stratification quantified individualized absolute survival benefits, culminating in a web‐based clinical decision‐support system.

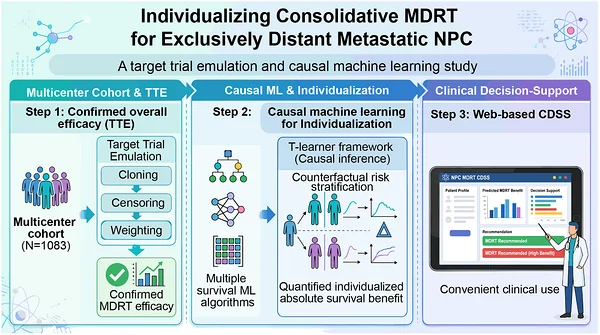

## 1 Introduction

Nasopharyngeal carcinoma (NPC) is a malignant epithelial tumor originating from the nasopharynx, with a distinct geographical distribution pattern [1]. Despite advances in intensity‐modulated radiation therapy (IMRT) and comprehensive treatment strategies incorporating chemotherapy and immunotherapy, which have significantly enhanced the therapeutic efficacy of NPC, distant metastasis remains one of the primary causes of treatment failure and mortality [2, 3]. Distant metastasis occurs in 15%–30% of patients with NPC following initial definitive treatment, with exclusive distant metastasis (defined as distant metastasis without synchronous locoregional recurrence) accounting for 55% of all treatment failures [4, 5, 6].

Recent trials have shown that the addition of PD‐1 inhibitors to first‐line chemotherapy significantly improves progression‐free survival (PFS) in patients with metastatic NPC [7, 8, 9]. Accordingly, NCCN and ESMO‐EURACAN guidelines recommend chemoimmunotherapy (CIT) as the first‐line standard of treatment [10, 11]. To further optimize survival benefits beyond systemic therapy alone, the integration of metastasis‐directed radiotherapy (MDRT) has been validated as an effective strategy across multiple studies [12, 13, 14, 15].

However, the existing evidence is predominantly derived from cohorts with de novo metastatic disease [16, 17]. For the clinically distinct subgroup presenting with exclusive distant metastasis, there is currently no clear consensus on the optimal therapeutic paradigm following initial systemic disease control. It remains unclear whether supplementing CIT with consolidative MDRT provides additional survival benefits for patients who achieve non‐progressive disease, and which patients would benefit from additional MDRT in the era of immunotherapy. Furthermore, retrospective evidence in this setting is frequently confounded by selection bias and immortal time bias. Consequently, no validated framework currently exists to estimate the individual absolute benefit of MDRT and optimize patient selection.

We hypothesized that the absolute survival benefit of MDRT would vary substantially according to individualized prognostic profiles. To test this hypothesis and address the current clinical dilemma, we conducted a multicenter study integrating a target trial emulation (TTE) design with a causal machine learning (ML) framework. TTE offers a rigorous analytic approach that incorporates core principles of randomized controlled trials into the design of observational studies [18, 19]. To establish the overall efficacy of MDRT, this method was employed to reduce both selection bias and immortal time bias [20, 21]. ML offers distinct advantages in capturing non‐linear interactions and identifying novel prognostic patterns [22]. Given the current absence of robust ML‐based prediction models for NPC with exclusive distant metastasis, we developed and validated an interpretable ML model. To move beyond risk prediction and answer counterfactual clinical questions, we applied a causal framework to estimate heterogeneous treatment effects (HTEs). Using this integrated framework, we identified marked heterogeneity in the model‐estimated benefit of MDRT and translated these estimates into an individualized clinical decision‐support system. By embedding this tool into clinical workflows, this study seeks to bridge the gap between PFS prediction and MDRT decision support, ultimately providing individualized benefit estimations to support personalized therapeutic strategies.

## 2 Results

### 2.1 Patient Characteristics

The flowchart of the study is shown in Figure 1. A total of 1083 patients with exclusive distant metastatic NPC who achieved non‐progressive disease following first‐line CIT formed the base TTE cohort. Within this cohort, MDRT was administered to 264 of 931 patients (28.4%) in the derivation cohort and 51 of 152 patients (33.6%) in the external validation cohort. The annual distribution of patients revealed a gradual increase in the proportion of MDRT administration (Figure S1A), reflecting its expanding clinical adoption in real‐world practice. The demographic and baseline characteristics of the base TTE cohort are summarized in Table 1. The median age of the overall cohort was 45 years (interquartile range [IQR], 37–54), and 78.5% (*n* = 850) were male. Lung was the most common metastatic site (40.7%), followed by bone (40.5%), and liver (34.3%). Multiple metastatic sites (> 2) were present in 227 (21.0%) patients, and metastatic lesions > 5 were observed in 730 (67.4%) patients. Following CIT within the TTE cohort, 764 (70.5%) patients achieved complete response (CR) or partial response (PR), while 319 (29.5%) patients exhibited stable disease (SD). The median equivalent dose in 2 Gy fractions (EQD2) for patients receiving MDRT was 52 Gy (41–60 Gy).

**FIGURE 1:**
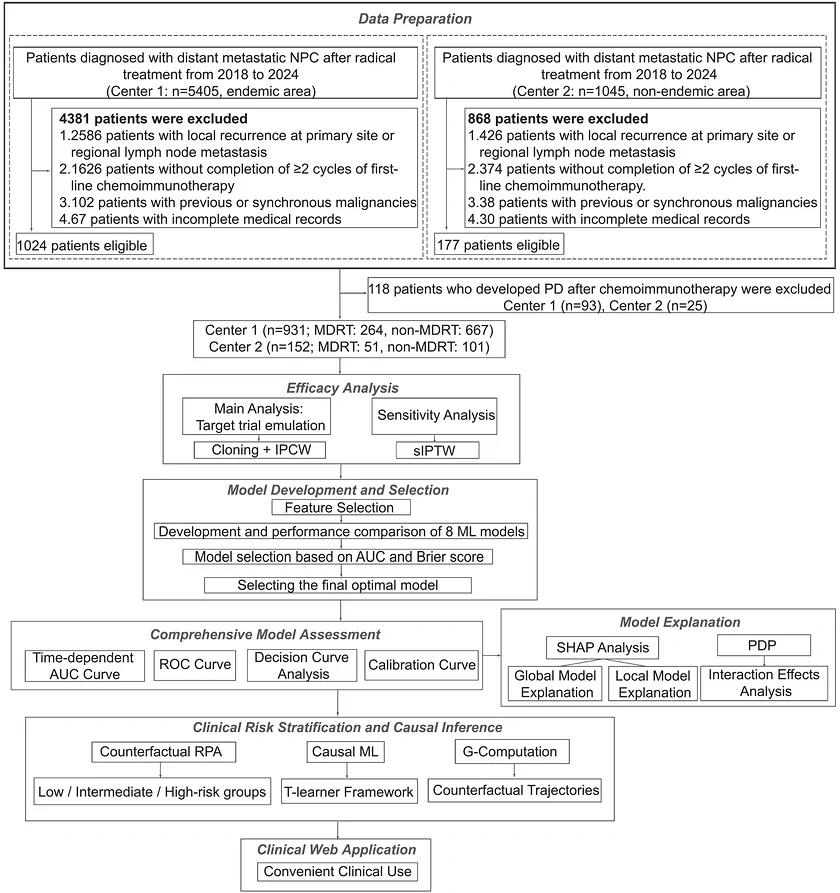
The study flowchart. AUC, area under the curve; MDRT, metastasis‐directed radiotherapy; ML, machine learning; PDP, partial dependence plots; ROC, receiver operating characteristic; RPA, recursive partitioning analysis; SHAP, Shapley additive explanations.

**TABLE 1:** Baseline demographic and clinical characteristics of the overall target trial population and by center.

Characteristics	Overall (*n* = 1083)	Derivation cohort (*n* = 931)	External validation cohort (*n* = 152)
Age, years	45 (37, 54)	45 (37, 53)	49 (41, 57)
Male, *n* (%)	850 (78.5)	726 (78.0)	124 (81.6)
Smoking, *n* (%)	330 (30.5)	275 (29.5)	55 (36.2)
Alcohol, *n* (%)	187 (17.3)	151 (16.2)	36 (23.7)
ECOG PS, *n* (%)			
0	934 (86.2)	809 (86.9)	125 (82.2)
1	149 (13.8)	122 (13.1)	27 (17.8)
Pre‐EBV DNA (copies/mL)	951 (132, 6100)	972 (133, 6160)	787 (123, 5575)
Pre‐CRP (mg/L)	2 (1, 11)	3 (1, 12)	1 (0, 5)
Pre‐LDH (U/L)	184 (162, 219)	184 (161, 220)	184 (168, 211)
Pre‐NLR	3.76 (2.59, 5.54)	3.89 (2.68, 5.68)	3.03 (2.29, 4.53)
Pre‐PLR	243 (174, 328)	248 (180, 336)	184 (138, 280)
Pre‐SII	845 (578, 1369)	861 (597, 1427)	721 (468, 1080)
Pre‐PNI	50.2 (47.3, 52.8)	50.0 (47.3, 52.7)	51.4 (48.3, 54.0)
Post‐EBV DNA (copies/mL)	0 (0, 20)	0 (0, 0)	0 (0, 372)
Post‐CRP (mg/L)	4 (1, 10)	4 (1, 10)	2 (1, 9)
Post‐LDH (U/L)	187 (164, 223)	187 (163, 222)	189 (167, 232)
Post‐NLR	4.0 (2.5, 6.3)	4.2 (2.6, 6.5)	3.1 (1.9, 4.7)
Post‐PLR	259 (170, 393)	273 (178, 408)	182 (146, 273)
Post‐SII	803 (475, 1376)	838 (492, 1446)	603 (390, 967)
Post‐PNI	48.3 (45.1, 51.1)	48.2 (45.0, 50.9)	49.3 (45.7, 52.0)
Liver metastasis, *n* (%)	372 (34.3)	319 (34.3)	53 (34.9)
Bone metastasis, *n* (%)	439 (40.5)	370 (39.7)	69 (45.4)
Lung metastasis, *n* (%)	441 (40.7)	372 (40.0)	69 (45.4)
Other metastasis, *n* (%)	341 (31.5)	305 (32.8)	36 (23.7)
No. of metastatic sites, *n* (%)			
≤ 2	856 (79.0)	729 (78.3)	127 (83.6)
> 2	227 (21.0)	202 (21.7)	25 (16.4)
No. of metastatic lesions, *n* (%)			
≤ 5	353 (32.6)	318 (34.2)	35 (23.0)
> 5	730 (67.4)	613 (65.8)	117 (77.0)
Cycles of CIT, *n* (%)			
< 6	397 (36.7)	341 (36.6)	56 (36.8)
≥ 6	686 (63.3)	590 (63.4)	96 (63.2)
MDRT, *n* (%)	315 (29.1)	264 (28.4)	51 (33.6)
Metastatic lesion response, *n* (%)			
CR	43 (4.0)	40 (4.3)	3 (2.0)
PR	721 (66.6)	624 (67.0)	97 (63.8)
SD	319 (29.5)	267 (28.7)	52 (34.2)

Abbreviations: CIT, chemoimmunotherapy; CR, complete response; CRP, C‐reactive protein; EBV, Epstein–Barr virus; ECOG PS, Eastern Cooperative Oncology Group performance status; LDH, lactate dehydrogenase; MDRT, metastasis‐directed radiotherapy; NLR, neutrophil‐to‐lymphocyte ratio; PLR, platelet‐to‐lymphocyte ratio; PNI, prognostic nutritional index; Post‐, post‐chemoimmunotherapy; PR, partial response; Pre‐, pre‐chemoimmunotherapy; SD, stable disease; SII, systemic immune‐inflammation index.

Before applying stabilized inverse probability of treatment weighting (sIPTW), the MDRT group exhibited baseline imbalances, presenting with a higher proportion of bone metastasis and a lower proportion of lung metastasis and post‐CRP levels. After sIPTW adjustment, the baseline characteristics were well‐balanced between the two groups (Table S1). The distribution of propensity scores showed substantial overlap between the two groups (Figure S2A), indicating balanced treatment allocation without extreme weighting inconsistencies. A visualization of the standardized mean difference (SMD) for each baseline covariate is shown in Figure S2B. Details of first‐line CIT and MDRT are provided in Tables S2 and S3, respectively. After multivariable adjustment, there was no significant difference in PFS among patients receiving various chemotherapy regimens or different immunotherapy drugs (Figure S3, Table S4). Furthermore, following sIPTW adjustment, post‐CIT maintenance strategies were balanced between the MDRT and non‐MDRT groups (Table S5).

### 2.2 Overall Survival Benefit of MDRT

At data cutoff, the median follow‐up was 32.5 months (95% confidence intervals [CIs], 29.4–35.5) in the MDRT group and 40.6 months (95% CI: 37.1–44.1) in the non‐MDRT group. A total of 56 patients (17.7%) in the MDRT group and 262 (34.1%) in the non‐MDRT group died. A total of 129 patients (40.9%) receiving MDRT and 490 patients (63.8%) not receiving MDRT had progressive disease (PD) or had died.

In the clone and inverse probability of censoring weights (IPCW) analysis (Figure 2), the MDRT group had a median PFS of 48.9 months (95% CI: 26.4–not reached [NR]), compared to 20.4 months (95% CI: 17.7–23.6) for the non‐MDRT group. The restricted mean survival time (RMST) difference for 3‐year PFS was 4.2 months (95% CI: 2.4–5.9, bootstrapped *p* < 0.001) with a Hazard ratio (HR) of 0.64 (95% CI: 0.52–0.77) (Figure 2A). The median overall survival (OS) was NR in the MDRT group, compared to 68.3 months (95% CI: 58.9–NR) in the non‐MDRT group (Figure S4A). The RMST difference for 3‐year OS was 1.5 months (95% CI: 0.1–2.9, bootstrapped *p* = 0.033) with an HR of 0.64 (95% CI: 0.45–0.86).

**FIGURE 2:**
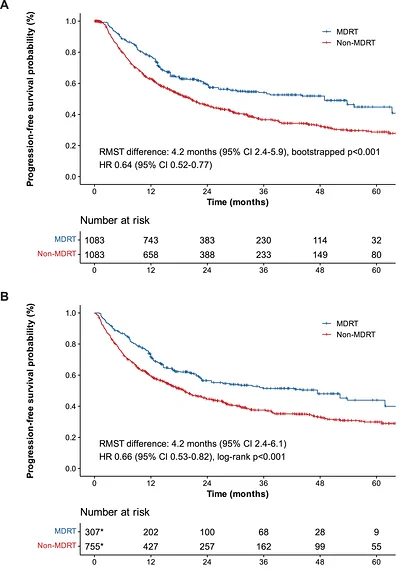
Kaplan–Meier curves of progression‐free survival after cloning‐censoring‐weighting (A) and sIPTW (B) analyses. MDRT, metastasis‐directed radiotherapy; sIPTW, stabilized inverse probability of treatment weighting. *The total number of patients in the two groups varies slightly in the post‐sIPTW pseudo‐data set due to weight truncation.

In the sIPTW population, the median PFS was 47.4 (95% CI: 22.8–NR) months for the MDRT group versus 19.0 months (95% CI: 16.2–22.9) for the non‐MDRT group (adjusted HR: 0.66 [95% CI: 0.53–0.82]; log‐rank *p* < 0.001; Figure 2B). In addition, the RMST difference for 3‐year PFS was 4.2 months (95% CI: 2.4–6.1). The OS was significantly longer in patients receiving MDRT than in those who did not (median, NR [95% CI: 61.9–NR] vs. 67.7 months [95% CI: 57.7–NR]; adjusted HR: 0.67, 95% CI: 0.48–0.93; log‐rank *p* = 0.008; Figure S4B). In addition, the overall findings remained consistent across multiple sensitivity analyses (Table S6, Figure S5).

The PFS benefit of MDRT was maintained across subgroups defined by metastatic burden (Figure S3). MDRT significantly reduced the risk of progression in both oligometastatic (HR: 0.66 [95% CI: 0.51–0.86]) and polymetastatic (HR 0.67 [95% CI: 0.46–0.97]) patients, with no significant interaction detected (*p*_interaction_ = 0.970). Within the MDRT cohort, unadjusted Kaplan–Meier analysis showed no significant difference in PFS between stereotactic body radiotherapy (SBRT) and IMRT (Figure S3, *p* = 0.89). Multivariable Cox regression further confirmed that the specific radiotherapy modality was not an independent prognostic factor for PFS (Table S7).

### 2.3 Development and Validation of the Prognostic Model

The feature selection process using the Boruta algorithm is illustrated in Figure 3A. The selection procedures for least absolute shrinkage and selection operator (LASSO), Random Survival Forest (RSF), and Elastic Net are presented in Figure S6. A total of seven overlapping predictors identified across all four methods were retained for model construction (Figure 3B), including pre‐EBV DNA, pre‐LDH, post‐EBV DNA, post‐CRP, number of metastatic lesions, metastatic lesion response, and MDRT. Based on the selected features, eight ML models for survival‐censored data were developed to predict dynamic survival (Table S8). Figure 3C presents a comparison of the CB score across different ML models. The gradient boosting machine (GBM) model achieved the highest CB score (0.812) after 10‐fold cross‐validation, outperforming all other algorithms and was selected as the final model for further evaluation.

**FIGURE 3:**
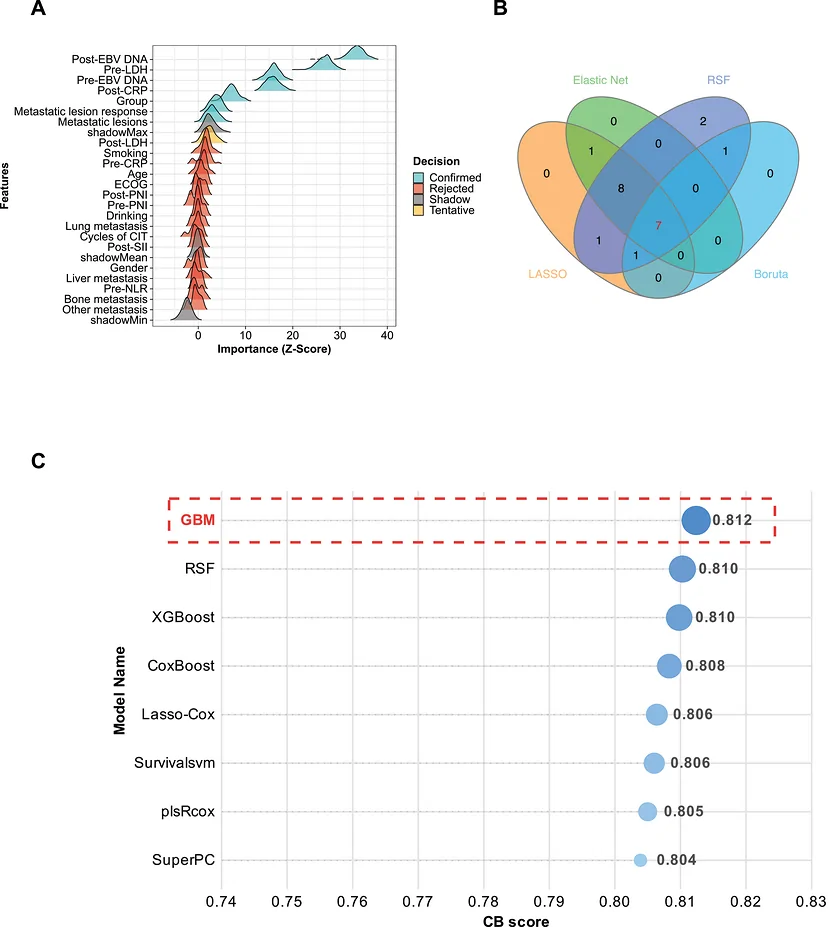
Feature selection and model performance comparison. (A) Feature importance ridge plot for variable selection based on Boruta. (B) Variable Venn diagram screened by four methods. MDRT was retained for the observed‐survival prediction model but was excluded from the baseline prognostic score used for subsequent RPA stratification. (C) Bubble plots for comparing machine learning model performance based on the CB score. CIT, chemoimmunotherapy; CoxBoost, Cox model with likelihood‐based boosting; CRP, C‐reactive protein; EBV, Epstein–Barr virus; ECOG, Eastern Cooperative Oncology Group; GBM, gradient boosting machine; LDH, lactate dehydrogenase; MDRT, metastasis‐directed radiotherapy; NLR, neutrophil‐to‐lymphocyte ratio; plsRcox, partial least squares Cox regression; PNI, prognostic nutritional index; Post‐, post‐chemoimmunotherapy; Pre‐, pre‐chemoimmunotherapy; RSF, random survival forest; SII, systemic immune‐inflammation index; SuperPC, super principal components; survivalsvm, survival support vector machine; XGBoost, extreme gradient boosting.

We evaluated the discriminative ability, calibration, and clinical utility of the optimal GBM model across both internal and external validation cohorts. To assess dynamic prediction performance, time‐dependent AUCs were generated (Figure 4A). The GBM model maintained robust discrimination for PFS over the 3‐year follow‐up, indicating stable temporal accuracy. For specific time‐point discrimination, the model yielded AUROCs of 0.843 (95% CI: 0.795–0.891), 0.853 (95% CI: 0.806–0.900), and 0.875 (95% CI: 0.826–0.924) for predicting 1‐, 2‐, and 3‐year PFS in the internal cohort, respectively (Figure 4B, Figure S7). This discriminatory capacity was successfully reproduced in the external cohort, with AUROCs of 0.819 (95% CI: 0.748–0.891), 0.844 (95% CI: 0.773–0.915), and 0.877 (95% CI: 0.804–0.949) at the respective milestones (Figure 4B, Figure S7). Calibration curves for 1‐, 2‐, and 3‐year PFS showed excellent agreement between the predicted probabilities and actual observations in both cohorts (Figure 4C,D). Furthermore, decision curve analysis (DCA) was utilized to evaluate the clinical utility (Figure 4E, Figure S7). Across a broad range of threshold probabilities, the GBM model consistently provided a higher net clinical benefit for predicting 1‐, 2‐, and 3‐year PFS compared to either the “treat‐all” or “treat‐none” strategies in both cohorts. The final model achieved an overall accuracy of 0.866 for 3‐year PFS in the external validation set (Figure 4F).

**FIGURE 4:**
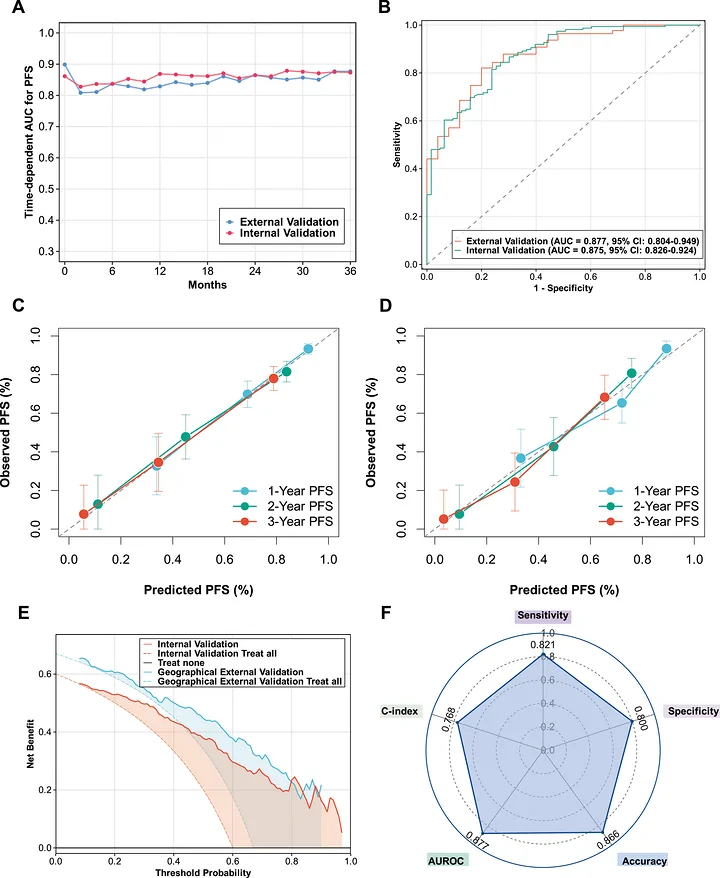
Model performance evaluation in the internal validation cohort and geographical external validation cohort. (A) Time‐dependent AUROC for PFS in the internal validation cohort and geographical external validation cohort. (B) ROC curves for predicting 3‐year PFS in the internal validation cohort and geographical external validation cohort. (C) Calibration curves for predicting 1‐, 2‐, and 3‐year PFS in the internal validation cohort. (D) Calibration curves for predicting 1‐, 2‐, and 3‐year PFS in the geographical external validation cohort. (E) Decision curve analysis for predicting 3‐year PFS in the internal validation cohort and geographical external validation cohort. (F) Radar plot of five key evaluation metrics for 3‐year PFS in the external validation set.

### 2.4 Model Interpretability and Feature Interaction Analysis

Multicollinearity analysis revealed that no substantial multicollinearity was detected among the variables. The maximum pairwise Spearman correlation across both cohorts was 0.239, and the maximum VIF was 1.70. The detailed metrics are provided in Table S9. The Shapley Additive Explanations (SHAP) framework was employed to evaluate the impact of variables on both the global and individual levels. Figure 5A illustrates the global feature importance, ordered from highest to lowest based on mean SHAP values, which establishes post‐EBV DNA, pre‐LDH, and post‐CRP as the leading prognostic factors. Furthermore, local explanations were applied to clarify how individual predictions are derived from patient‐specific feature values. Corresponding visualizations, including force plots, decision plots, and waterfall plots, are presented in Figure S8.

**FIGURE 5:**
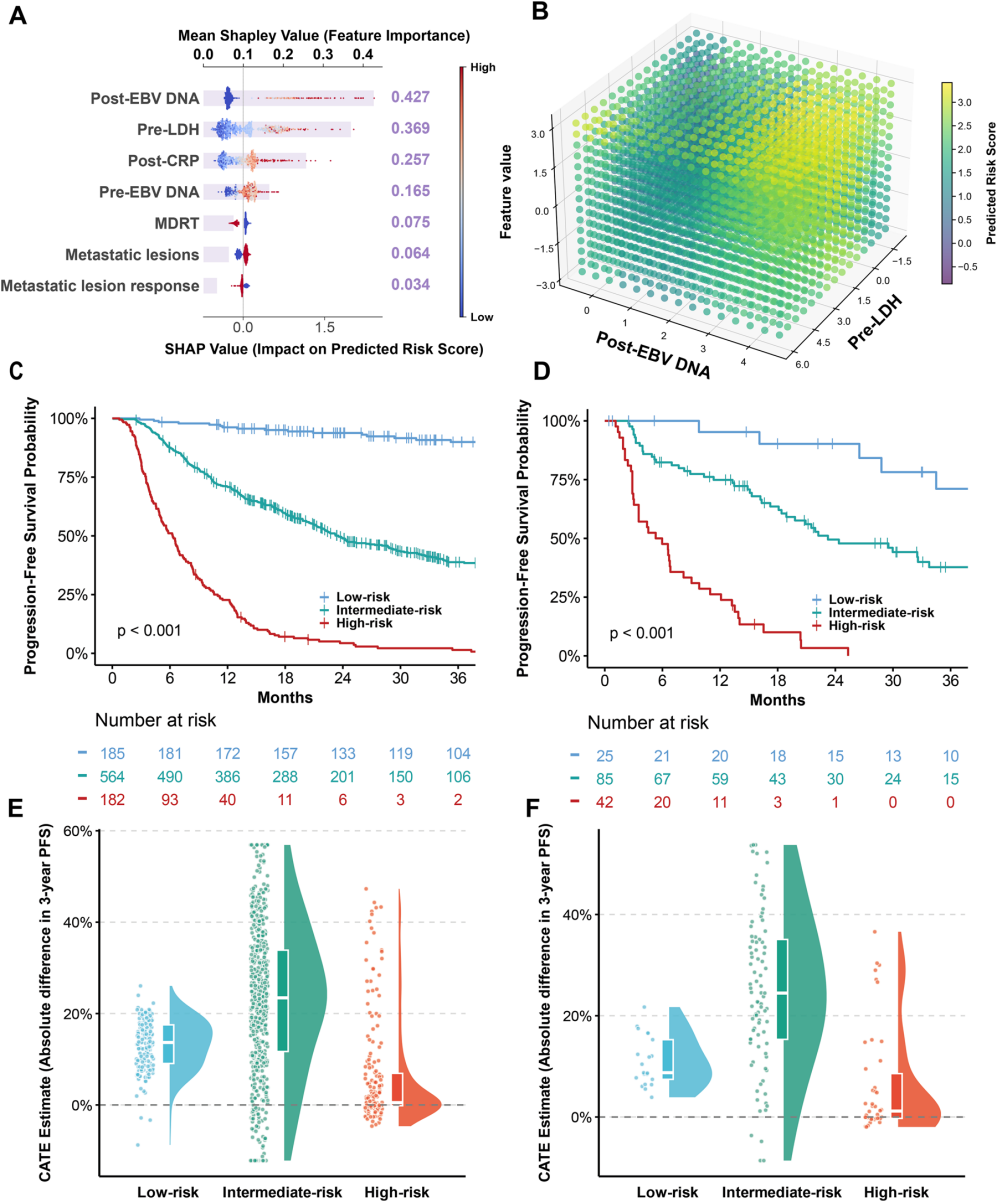
Model interpretability and causal inference analysis. (A) Evaluation of the contribution of each feature to the model using mean SHAP values, displayed in descending order, with color indicating feature value (red = high, blue = low) by SHAP summary plot (bee swarm). (B) Visualization of the joint effect of post‐EBV DNA, post‐CRP, and pre‐LDH on the predicted probability. Color intensity ranging from dark to light represents the predicted probability of progression, with lighter colors indicating higher risk by three‐dimensional partial dependence plot (3D PDP). (C, D) Kaplan–Meier curves for PFS across different risk groups in the derivation cohort (C) and external validation cohort (D). (E, F) Distribution of individual CATE of MDRT on 3‐year PFS in the derivation cohort (E) and external validation cohort (F). ARR, absolute risk reductions; CATE, conditional average treatment effects; CRP, C‐reactive protein; EBV, Epstein–Barr virus; LDH, lactate dehydrogenase; MDRT, metastasis‐directed radiotherapy; post‐, post‐chemoimmunotherapy; pre‐, pre‐chemoimmunotherapy.

A SHAP interaction heatmap was constructed to explore potential interactions among variables (Figure S9), highlighting prominent self‐interaction values for post‐EBV DNA, pre‐LDH, and post‐CRP. We utilized one‐dimensional partial dependence plots (1D‐PDPs) to evaluate the marginal effects of these specific variables on the predicted risk of disease progression (Figure S9), while their combined effects were captured in a three‐dimensional (3D) PDP (Figure 5B). The plot demonstrated that elevated pre‐LDH and high post‐EBV DNA levels were jointly associated with a higher predicted risk of progression. This joint nonlinear association was most pronounced in patients with concurrently high post‐CRP levels, indicating that the combination of these three elevated biomarkers compounds the progression risk.

### 2.5 Individualized Absolute Benefit of MDRT

Identifying the optimal candidates for MDRT is crucial for individualized management in clinical practice. To establish a natural prognostic baseline devoid of the MDRT effect, patients were stratified into low‐, intermediate‐, and high‐risk groups using recursive partitioning analysis (RPA) based on counterfactual risk scores (Table S10, Figure S10). Kaplan–Meier survival analysis revealed significant separation of PFS curves across the three risk groups in both the derivation and external validation cohorts (Figure 5C,D), confirming the excellent prognostic discrimination of baseline stratification.

We subsequently sought to estimate the individualized causal benefit of MDRT. Using the established T‐learner framework, we estimated the conditional average treatment effects (CATE) for 1‐, 2‐, and 3‐year PFS, operationalized as the absolute risk reductions (ARR) (Figure S11). The T‐learner framework estimated the largest absolute treatment benefit for the intermediate‐risk group, with a mean 3‐year ARR of 20.9% in the derivation cohort and 21.6% when the locked models were applied to the external cohort. The low‐risk group also exhibited clinically meaningful benefits with corresponding model‐estimated ARRs of 15.8% and 14.7%, respectively. Conversely, the high‐risk group obtained only marginal absolute benefits (4.9% and 5.7%, respectively) (Figure 5E,F). As a robustness check, a sensitivity analysis refitting the baseline score without censoring weights yielded almost identical rankings (Spearman's *ρ* = 0.99 in both cohorts), assigned the same risk tier for 92% of patients (Table S11), and demonstrated an unchanged gradient of absolute benefit across tiers (Table S12). Furthermore, to ensure the reliability of these estimations, both arm‐specific T‐learner models were well calibrated. In the derivation cohort, the mean predicted and observed 3‐year PFS differed by less than 3 percentage points in each arm (MDRT 52.7% vs. 55.4%, and non‐MDRT 36.0% vs. 37.9%). The models demonstrated good quintile‐level agreement (Figure S12). This calibration accuracy was successfully maintained in the external cohort (MDRT 54.4% vs. 50.7%, and non‐MDRT 27.0% vs. 25.0%).

### 2.6 Counterfactual Survival Trajectories

To visualize the temporal evolution of the model‐estimated treatment effects, stratified G‐computation was used to reconstruct marginal expected PFS trajectories under the two hypothetical treatment strategies in the external validation cohort (Figure 6). In the intermediate‐ and low‐risk groups, the model‐predicted trajectories showed progressively widening separation between the MDRT and non‐MDRT strategies. At 3 years, the corresponding model‐estimated absolute differences were 21.6% and 14.7%, respectively. In contrast, the predicted trajectories in the high‐risk group largely overlapped, corresponding to a model‐estimated 3‐year ARR of 5.7%.

**FIGURE 6:**
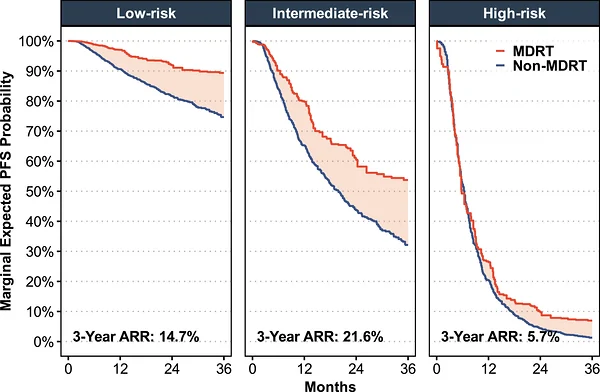
Counterfactual marginal expected PFS under MDRT versus non‐MDRT across different risk groups. MDRT, metastasis‐directed radiotherapy; PFS, progression‐free survival.

Finally, sensitivity analyses were conducted to assess the robustness of the estimated causal effects. Random Gaussian noise (5%) was introduced into the continuous clinical variables, and the individualized CATEs were re‐estimated, demonstrating high consistency with the original predictions (Table S13). Furthermore, sensitivity to unmeasured confounding was quantified, yielding *E*‐values that consistently confirmed the robustness of the observed survival benefits against potential unmeasured confounding across all risk groups (Table S13).

### 2.7 Accessible Web Application for Clinical Utility

To translate our findings into clinical practice, we developed an interactive web‐based clinical decision support system (CDSS) integrating the established T‐learner causal framework (Figure S13). This CDSS is intended for use at the precise clinical juncture of tumor response evaluation after CIT (the individualized time zero [*T*_0_]). Users can input the required feature values under the relevant module, and the application will automatically compute the individualized PFS at 1‐, 2‐, and 3‐year milestones, directly comparing the projected outcomes of maintaining systemic therapy alone versus the addition of MDRT. Furthermore, the tool calculates the 3‐year ARR, assigns the patient to a specific risk group, and provides individualized prognostic estimates regarding the potential benefit of MDRT. The web application can be accessed online at the following link: https://npc‐mdrt‐decision‐support.shinyapps.io/npc_decision_support/.

To ensure safe clinical implementation, the web‐based CDSS is programmed to mandate all required predictor fields. The application will proactively prompt a warning message and halt the survival prediction if any input data is missing or falls outside biologically plausible ranges.

## 3 Discussion

While integrating MDRT with CIT shows clinical promise, optimal patient selection for consolidative MDRT among non‐progressive patients following CIT with exclusively distant metastatic NPC remains a critical dilemma. To our knowledge, this study represents the largest cohort analysis to date evaluating MDRT efficacy in this specific population across both endemic and non‐endemic regions, as well as the first to develop a digital decision‐support system integrating TTE with a causal ML framework. Our analyses suggest that the model‐estimated absolute PFS benefit associated with MDRT varies across baseline prognostic risk groups, yielding substantial gains for low‐ and intermediate‐risk patients while offering marginal value to high‐risk cohorts. By translating complex counterfactual inferences into an accessible web‐based tool, this study bridges the gap between robust causal methodology and real‐world clinical utility, shifting the management of metastatic disease toward individualized, data‐driven treatment decisions.

While incorporating MDRT into systemic treatments improves survival in de novo metastatic NPC [12, 23], its specific efficacy as a consolidative strategy within the distinct subgroup of exclusively distant metastasis remains undefined, and retrospective evidence is frequently confounded by immortal time bias and other design flaws [16, 24]. Using the TTE framework to minimize these biases, we demonstrated that adding MDRT significantly improved both PFS and OS, a finding consistently supported by multiple sensitivity analyses. Radiotherapy can induce immunogenic cell death and upregulate PD‐L1 expression on tumor and inflammatory cells, providing a basis for synergy with immunotherapy [25, 26, 27, 28]. Our findings contrast with the recent CAMRESBRT trial, which reported no survival benefit when adding SBRT to camrelizumab in recurrent or metastatic NPC [29]. The CAMRESBRT trial enrolled a heterogeneous population including locoregional recurrences, relied on PD‐1 monotherapy, and irradiated only a single metastatic site, whereas our study selectively included patients with exclusive distant metastasis who had achieved non‐progressive status following CIT and prioritized comprehensive residual‐lesion coverage. Local radiotherapy efficacy thus depends heavily on precise patient selection, effective systemic therapy, and maximum cytoreduction rather than the simple addition of localized radiation.

Utilizing the SHAP framework, our model identified post‐CIT EBV DNA and CRP, as well as pre‐CIT LDH, as key factors driving disease progression. Dynamic changes in plasma EBV DNA have been consistently associated with response to chemotherapy and immunotherapy [30, 31], and early EBV DNA clearance predicted longer PFS in the CAPTAIN‐1st study [32]. Biologically, these biomarkers reflect residual tumor burden, metabolic aggressiveness, and systemic inflammation, respectively [33, 34]. The ML approach further captures high‐dimensional, non‐linear synergies [35], as illustrated by the 3D‐PDP, where concurrently elevated EBV DNA and LDH compound the risk of progression beyond either marker alone. With minimal collinearity among the retained features (all pairwise Spearman coefficients < 0.3) and consistent selection across four screening algorithms, these SHAP rankings should be interpreted jointly as capturing complementary dimensions of tumor burden and systemic inflammation. Clinically, although these patients achieved macroscopic disease control during initial CIT, their persistently high biomarkers indicate substantial minimal residual disease and ongoing systemic inflammation [34]. In such cases, survival is primarily threatened by systemic progression rather than local failure, and thus adding MDRT provides negligible benefit.

The critical clinical question is whether a specific strategy can actually alter a patient's disease trajectory and inform actionable interventions [36]. Unlike traditional ML, which primarily forecasts the risk of progression, causal ML estimates the individualized absolute benefit of adding a specific intervention under explicit causal assumptions [37]. Applying this principle through a T‐learner framework, we observed model‐estimated heterogeneity in the absolute PFS benefit of MDRT, with larger estimated ARRs in the low‐ and intermediate‐risk groups across both cohorts. Relying solely on relative measures like HRs frequently obscures the true absolute benefit [16]. For intensive localized interventions such as MDRT, establishing a clear absolute survival benefit justifies aggressive local therapy on an individual basis, preventing the undertreatment of optimal candidates.

In clinical practice, the developed web‐based CDSS translates these causal inferences into intuitive, quantitative references. Designed to complement multidisciplinary team (MDT) evaluations rather than replace clinical judgment, the CDSS highlights low‐ and intermediate‐risk patients as potential MDRT candidates. For the high‐risk group, when the CDSS estimates a relatively small potential benefit from MDRT, this information may serve as one component of multidisciplinary decision‐making and may prompt consideration of alternative systemic or investigational strategies, such as intensified systemic regimens, novel immunotherapy combinations, or early enrollment in clinical trials.

In the absence of a formally established minimum clinically important difference for consolidative MDRT, we propose a 3‐year ARR of 10% as a pragmatic decision‐support benchmark. Although local radiotherapy added to systemic therapy yielded absolute OS gains of roughly 22% at 2 years in metastatic NPC [38] and 25% at 5 years across solid tumors [39], expecting such large benefits for individual decision‐making would be overly restrictive. The conservative 10% benchmark aligns with the highest benefit grade of the ESMO Magnitude of Clinical Benefit Scale (ESMO‐MCBS) in the non‐curative setting [40]. Conversely, an estimated benefit below 5% generally discourages the addition of MDRT. Based on the threshold model of clinical decision‐making, intervention is justified only when expected benefit exceeds expected harm [41]. Contemporary MDRT techniques carry a severe toxicity rate of approximately 5% [42]. The 5%–10% interval therefore forms a preference‐sensitive gray zone, and we frame these thresholds as anchors for shared decision‐making rather than rigid deterministic rules. Crucially, these thresholds apply to individual patient estimates rather than risk‐tier averages, which would discard valuable personalized data and undermine the advantage of the CDSS.

Treatment selection for this population has often relied on baseline tumor burden [43], a static approach that fails to capture dynamic responses to first‐line systemic therapy. By quantifying treatment benefits based on longitudinal profiles across the CIT course, our model shifts disease management toward a response‐guided strategy. Furthermore, previous prognostic models for NPC are often constrained by single‐center designs or data restricted entirely to endemic regions [44]. Validating our framework across both endemic and non‐endemic cohorts ensures it accounts for geographic variations in EBV biology and institutional treatment practices.

Despite the promising results of this study, several limitations warrant consideration. First, despite the TTE framework and comprehensive sensitivity analyses, residual bias and unmeasured confounding remain due to the retrospective nature of this study. Second, because the cohorts consisted exclusively of Chinese patients, the model's generalizability to other ethnic populations needs further validation. Third, while the current model relies on routinely accessible clinical and biomarker profiles, it does not include deeper molecular profiling data. Future models incorporating multi‐omics data are warranted to further refine these predictions. Fourth, detailed data on treatment‐related toxicities were not accurately captured, and immunotherapy agents and radiotherapy techniques varied across participating centers. Finally, although the prognostic component was externally validated and the treatment‐benefit models were transported to an independent cohort, the individualized treatment‐effect estimates require further validation in independent prospective data.

## 4 Conclusion

This study developed the first causal ML framework to estimate the individualized survival benefit of consolidative MDRT in exclusively distant metastatic NPC. By translating these estimates into an accessible CDSS, this study provides a quantitative reference to identify optimal candidates for MDRT and support multidisciplinary clinical decision‐making, thereby facilitating the precision management of metastatic disease.

## 5 Methods

### 5.1 Study Design and Patient Selection

This multicenter study included 1083 NPC patients who developed distant metastasis following definitive treatment across two independent hospitals. The derivation cohort consisted of 931 patients treated at the Sun Yat‐sen University Cancer Center (SYSUCC) from January 2018 to December 2024. The geographical external cohort included 152 patients at Cancer Hospital, Chinese Academy of Medical Sciences (CAMS) during the same period. The inclusion criteria were: (1) Evidence of exclusively distant metastasis, defined as distant metastasis without synchronous locoregional recurrence (no recurrence at the primary site or regional lymph nodes) at the time of metastasis diagnosis, based on a comprehensive evaluation via pathology or imaging; (2) previously undergone definitive treatment for the primary tumor; (3) received at least two cycles of first‐line CIT; (4) age 18 years or older; (5) Eastern Cooperative Oncology Group performance status (ECOG PS) of 0–1. The exclusion criteria were: (1) Incomplete medical records or follow‐up documentation; (2) synchronous local recurrence at the primary site or regional lymph node metastasis at the time of distant metastasis diagnosis; (3) PD at any time during first‐line CIT or at the initial post‐CIT efficacy evaluation; (4) combined with other malignancies. This study was conducted and reported in accordance with the STROBE guidelines for observational studies [45].

### 5.2 Treatment

At each center, a MDT determined treatment plans combining systemic and local therapies. Chemotherapy regimens and immunotherapy agents are listed in the Supporting Information. In the MDRT group, the intervention was defined as a pragmatic strategy, systematically determined following the efficacy evaluation of first‐line CIT. For oligometastatic patients (number of metastatic sites ≤ 2; number of metastatic lesions ≤ 5), consolidative radiotherapy aiming at the ablation of all residual lesions was prioritized. For non‐oligometastatic patients, MDRT was delivered as a cytoreductive or consolidative strategy to maximize local tumor control and delay progression. Radiotherapy techniques, prescribed doses, and fractionation schedules are described in the Supporting Information.

### 5.3 Follow‐Up and Outcome

Patients underwent regular assessments every two to three cycles during CIT and every 3 months post‐completion of all treatments. Each follow‐up consisted of physical assessment and imaging with CT or MRI. Evaluations were conducted by two experienced radiologists and a skilled radiation oncologist, following the Response Evaluation Criteria in Solid Tumors (RECIST 1.1). The primary endpoint was PFS, defined as the time from T_0_ to the first documented disease progression or death from any cause. The secondary endpoint was OS, defined as the time from T_0_ to death from any cause. For both endpoints, event‐free patients or those lost to follow‐up were censored at the date of their last contact.

### 5.4 Target Trial Emulation

We adopted a TTE framework to minimize selection bias, confounding, and immortal time bias, following the TARGET reporting statement [19]. The protocol of the hypothetical target trial and its emulation is summarized in Table S15. The T_0_ was defined as the date of the first efficacy evaluation upon the completion or termination of first‐line CIT. Although up to six cycles are typically recommended [46], this initial systemic phase may conclude earlier based on MDT assessment of clinical factors, such as individual treatment tolerance and optimal clinical response. Consequently, T_0_ serves as a uniform decision node marking the conclusion of first‐line CIT, at which point the addition of consolidative MDRT is considered. Patients experiencing disease progression or dying prior to T_0_ were excluded from the analytical cohort. A cloning–censoring–weighting approach akin to an intention‐to‐treat (ITT) analysis was adopted [47]. At T_0_, each eligible patient was cloned and assigned to both strategies under evaluation, namely initiation of consolidative MDRT within a 1.5‐month grace period or no initiation within the same window. The grace period was defined from standard clinical workflows, in which 2–6 weeks typically elapse between post‐CIT response evaluation and MDRT delivery (Figure S1B), and alternative grace periods of 1.0 and 2.0 months were assessed in sensitivity analyses. Clones deviating from their assigned strategy were artificially censored at the time of deviation, whereas patients who progressed or died during the grace period contributed events in both arms. To address potential selection bias introduced by artificial censoring, arm‐specific logistic regression models on the baseline covariates were fitted to estimate each clone's probability of remaining uncensored, and unstabilized IPCW were applied from the end of the grace period onward, with symmetric truncation at the 1st and 99th percentiles (Table S16). Because the cloning step inherently violates the proportional hazards assumption, RMST was utilized as the primary metric to assess differences in PFS and OS. HRs from weighted Cox proportional hazards models were additionally calculated for reference. To account for the artificially inflated sample size generated by the cloning procedure, 95% CIs were computed using non‐parametric bootstrapping with 1000 resamples.

### 5.5 Prognostic Model Development, Comparisons, and Evaluation

The derivation cohort was randomly split into a training set (70%) and an internal validation set (30%), and all feature selection and preprocessing procedures were conducted solely within the training set to prevent data leakage. After removing redundant variables with pairwise correlation coefficients of 0.6 or higher, four algorithms (Boruta, LASSO, RSF, and Elastic Net) were applied, and only variables consistently selected by all four methods were retained following review by senior NPC specialists. Eight machine learning algorithms adapted for censored survival data were developed to predict progression risk, including LASSO Cox regression, RSF, Extreme Gradient Boosting (XGBoost), GBM, Cox model with likelihood‐based boosting (CoxBoost), Survival Support Vector Machine (Survivalsvm), Super Principal Components (SuperPC), and Partial Least Squares Cox regression (plsRcox). A 1.5‐month conditional landmark analysis was applied to the ML cohort, excluding patients who progressed or died (*n* = 8) or were lost to follow‐up (*n* = 2) within the landmark and leaving 1073 patients (925 in the derivation cohort and 148 in the external cohort) for model development and validation. To select the optimal prediction model, we defined a composite metric, the CB score, which combines the integrated Area Under the Receiver Operating Characteristic Curve (iAUC) and the Integrated Brier Score (IBS) to simultaneously assess both the discriminative and calibration abilities of each algorithm [48]. The performance of the optimal model was evaluated in both the internal validation set and the geographical external validation set. Discrimination was assessed via time‐dependent AUCs, specifically evaluating the 1‐, 2‐, and 3‐year PFS. The RCS transformation parameters, including knots and coefficients, were derived solely from the training set and applied as a locked function to both validation cohorts. Calibration at these milestones was then assessed using the Kaplan–Meier method with 1000 bootstrap resamples. Finally, DCA quantified the clinical net benefit of the model across continuous threshold probabilities at these specific time points. The development, validation, and reporting of the prediction model and the CDSS followed the TRIPOD+AI statement [49].

### 5.6 Model Explanation and Causal Inference Analysis

To interpret the decision‐making process of the ML algorithms, SHAP, a game‐theoretic approach, was employed to quantify the direction and magnitude of individual feature contributions to the predicted risk. Building upon the established TTE design, a counterfactual causal inference framework was implemented to systematically investigate the HTE of MDRT on PFS. To prevent data leakage, all causal model derivations and threshold definitions were exclusively conducted in the derivation cohort and subsequently projected onto the external validation cohort. First, within the derivation cohort, a covariate‐adjusted baseline prognostic score was generated by fitting the optimal ML model exclusively within the non‐MDRT arm of the emulated trial. This score represents the counterfactual prognosis had no patient received consolidative MDRT. A 10‐fold cross‐validated RPA survival tree then stratified patients into three risk groups. A T‐learner framework was subsequently deployed to estimate individualized CATE and ARR. Two independent optimal ML models were trained separately on the treated and untreated pseudo‐populations. These models were applied across both cohorts to compute individual counterfactual survival differences. Marginal expected survival trajectories under each strategy were reconstructed via G‐computation, with individual conditional survival curves averaged over the empirical covariate distribution within each risk group. Finally, sensitivity analyses were conducted to assess the robustness of the causal estimates against measurement error and unmeasured confounding. Implementation details are provided in the Supporting Information.

### 5.7 Statistical Analysis

Descriptive statistics were utilized to summarize baseline patient characteristics. The normality of continuous variables was evaluated using the Kolmogorov–Smirnov test; normally distributed variables were expressed as means with standard deviations, whereas skewed variables were reported as medians with IQR. Categorical variables were compared using the χ^2^ test or the Fisher exact test. Continuous variables were compared using the Mann–Whitney *U* test or independent‐samples *t*‐test. Continuous variables underwent logarithmic transformation for skewed distributions followed by *Z*‐score normalization, while categorical variables were processed via label and ordinal encoding. To preclude data leakage, all preprocessing parameters were computed solely from the training set (for internal validation) or the derivation cohort (for external validation), and prospectively applied to transform the unseen validation sets. A significance level of *p* ≤ 0.05 was considered statistically significant in all analyses. All analyses were performed with R (version 4.2.2) and Python (version 3.8.2).

## Author Contributions

M.S. and M.D. were responsible for project administration, conceptualization, and investigation of the study, and also reviewed and edited the manuscript. X.W., S.C., and X.Z. contributed to methodology development and drafting of the original manuscript. L.M., J.X., and Y.Z. contributed to formal analysis and data validation. X.W., J.C., S.Y., and X.G. were involved in data curation. Z.D., M.D., M.S., L.Z., and S.Z. provided supervision for the study. M.S. and Z.D. had full access to all data in the study and took responsibility for the integrity of the data. The corresponding authors assumed final responsibility for the decision to submit the manuscript. All authors reviewed and approved the final manuscript.

## Funding

This work was supported by the National Natural Science Foundation of China (82403181), the Natural Science Foundation of Guangdong Province (Basic and Applied Basic Research Foundation of Guangdong Province) (2023A1515110258), and the Natural Science Foundation of Guangdong Province (Basic and Applied Basic Research Foundation of Guangdong Province) (2024A1515013244).

## Ethics Statement

This study was conducted in accordance with the Declaration of Helsinki and approved by the Institutional Review Boards of SYSUCC (B2026‐436‐01) and CAMS (NCC6477). All patient data were anonymized prior to analysis, and the requirement for written informed consent was waived by the Institutional Review Boards of all participating centers due to the retrospective nature of the study.

## Conflicts of Interest

The authors declare no conflicts of interest.

## Supporting information




**Supporting Information file 1**:mco271089‐sup‐0001‐SuppMat.pdf

## Data Availability

Study data are available on the Research Data Deposit public platform (www.researchdata.org.cn). Data sharing requests should be sent to the corresponding author. The code is available on GitHub. (GitHub—wangxg888/NPC‐CausalML‐MDRT: Code for survival analysis and causal machine learning framework).
