# Supplementary material for: Estimating Individualized Survival Benefit of Consolidative Metastasis‐Directed Radiotherapy Following Chemoimmunotherapy in Nasopharyngeal Carcinoma With Exclusive Distant Metastasis
Source: MedComm (2020). 2026 Oct 23;7(11):e71089. doi: 10.1002/mco2.71089 (PMC13693920; doi:10.1002/mco2.71089)
Supplement: Supplementary file 1 [file MCO2-7-e71089-s001.pdf]

Supplementary Materials for

Estimating individualized survival benefit of consolidative metastasis-directed radiotherapy following chemoimmunotherapy in nasopharyngeal carcinoma with exclusive distant metastasis

Xuguang Wang[1, 2, #], MD; Shuwei Chen[1, 2, 3, #], MD; Xing Zhang[1, 2, 3, #], MD; Linfei Mao[1, 2, 3], MD; Jingtao Chen[1, 2, 3], MD; Shida Yan[1, 2, 3], MD; Jiawei Xie[1, 2, 3], MD; Xianlu Gao[1, 2, 3], MD; Liji Zheng[1, 2, 3], MD; Yujie Zeng[1, 2, 3], MD; Shiting Zhang[1, 2, 3], MD; Zekun Deng[4, *], MD; Mingyuan Du[1, 2, 3, *], MD; Ming Song[1, 2, 3, *], MD.

Affiliations:
[1]State Key Laboratory of Oncology in South China, Guangzhou, China.
[2]Guangdong Provincial Clinical Research Center for Cancer, Guangzhou, China.
[3]Department of Head and Neck Surgery, Sun Yat-sen University Cancer Center, Guangzhou, China.
[4]Department of Neurosurgery, National Cancer Center/National Clinical Research Center for Cancer/Cancer Hospital, Chinese Academy of Medical Sciences and Peking Union Medical College, Beijing, China.
[#] These authors contributed equally.
* These authors jointly supervised this work.

Corresponding authors:
Dr Ming Song: Department of Head and Neck Surgery, Sun Yat-sen University Cancer Center, State Key Laboratory of Oncology in South China, Collaborative Innovation Center for Cancer Medicine, 510060, Guangzhou, Guangdong, P. R. China. E-mail addresses: songming@sysucc.org.cn
Dr Mingyuan Du: Department of Head and Neck Surgery, Sun Yat-sen University Cancer Center, 651 Dongfengdong Road, Guangzhou, 510060, China. E-mail addresses: dumy@sysucc.org.cn
Dr Zekun Deng: Department of Neurosurgery, National Cancer Center/National Clinical Research Center for Cancer/Cancer Hospital, Chinese Academy of Medical Sciences and Peking Union Medical College, Beijing, China. E-mail addresses: zekundeng@student.pumc.edu.cn

Contents

Supplemental Methods

Supplementary Methods 1. Treatment details....4
Supplementary Methods 2. Data collection and quality control....4
Supplementary Methods 3. Target trial emulation specifications....5
Supplementary Methods 4. Sample size estimation....6
Supplementary Methods 5. Sensitivity analyses....6
Supplementary Methods 6. Feature selection and model development details....7
Supplementary Methods 7. Causal inference implementation details....8

Supplementary Figures

Figure S1. Annual distribution of patients by treatment group and density and distribution of time from tumor response evaluation (T0) to the initiation of MDRT....9
Figure S2. The distribution of propensity scores and standardized mean differences after applying sIPTW adjustments....10
Figure S3. Kaplan-Meier estimates of progression-free survival stratified by systemic treatment regimens and radiotherapy modalities and subgroup forest plot stratified by metastatic burden....11
Figure S4. Kaplan–Meier curves of overall survival after cloning-censoring-weighting and sIPTW analyses....12
Figure S5. Kaplan-Meier curves of progression-free survival and overall survival across sensitivity analyses....13
Figure S6. Variable selection using Least Absolute Shrinkage and Selection Operator, Random Survival Forest, and Elastic Net....15
Figure S7. Model performance evaluation in the internal validation cohort and geographical external validation cohort....16
Figure S8. Local SHAP explanations for representative samples....17
Figure S9. SHAP interaction heatmap and SHAP dependence plots of key predictive features....18
Figure S10. Identification of prognostic risk groups using recursive partitioning analysis....19
Figure S11. Time-varying CATE of MDRT across prognostic risk groups....20
Figure S12. Calibration curves of the arm-specific T-learner models for predicting 3-year progression-free survival....21
Figure S13. Web-based clinical decision-support platform for individualized progression-free survival prediction and radiotherapeutic benefit estimation in metastatic nasopharyngeal carcinoma....22
Figure S14. Directed acyclic graphs used to identify confounding covariates for the survival adjustment sets in this study....23
Figure S15. Correlation filtering of highly correlated variables....24

Supplementary Tables

Table S1. Patient baseline characteristics before and after applying stabilized inverse probability of treatment weighting....26
Table S2. Details of first-line chemoimmunotherapy....27

Table S3. Details of metastasis-directed radiotherapy..28
Table S4. Univariable and multivariable analysis of progression-free survival assessing the impact of local and systemic treatment variations..............................29
Table S5. Patterns and sIPTW-weighted balance of post-chemoimmunotherapy maintenance strategies...30
Table S6. E-value Sensitivity Analyses for Unmeasured Confounding on Survival Outcomes within the Target Trial Emulation Framework..31
Table S7. Univariable and multivariable analysis of progression-free survival among patients receiving consolidative MDRT..32
Table S8. Hyper-parameters for the eight machine learning algorithms..................33
Table S9. Multicollinearity analysis of the final selected features in the machine learning models...34
Table S10. Stratification of counterfactual baseline risk driven by recursive partitioning analysis...35
Table S11. Sensitivity analysis of baseline risk stratification consistency after omitting censoring weights..36
Table S12. Mean estimated absolute risk reduction (ARR) with MDRT by risk tier and scoring approach..37
Table S13. Estimated overall and conditional treatment effects of MDRT on 3-year progression-free survival with robustness and sensitivity analyses..........................38
Table S14. Features used for machine learning algorithms development.................39
Table S15. Summary of the protocol for the hypothetical randomized trial and target trial emulation (Population-level survival analysis)..40
Table S16. Specification and distribution parameters of the inverse probability of censoring weighting in the target trial emulation..45
Table S17. Sample sizes and hyperparameter specifications of the arm-specific gradient boosting machine models within the T-learner framework in the derivation cohort...46
References

Supplemental Methods

Supplementary Methods 1. Treatment details

The standard chemotherapy regimens included GP (gemcitabine and platinum), TP (docetaxel/paclitaxel and platinum), PF (platinum and 5-fluorouracil), TPF (docetaxel/paclitaxel, platinum, and 5-fluorouracil), and TPC (docetaxel/paclitaxel, platinum, and capecitabine), typically administered over 2–6 cycles. Commonly used immunotherapy agents included camrelizumab, toripalimab, tislelizumab, pembrolizumab, penpulimab, sintilimab, and nivolumab. The radiotherapy modalities and fractionation schedules were individualized based on the lesion's size, location, and proximity to critical organs at risk. Prescribed doses typically ranged from 24 to 68.1 Gy, administered in 3 to 35 fractions, and adherence to dose constraints was prioritized over the prescribed dose in all cases. The radiotherapy techniques employed were intensity-modulated radiation therapy (IMRT) and stereotactic body radiation therapy (SBRT).

Supplementary Methods 2. Data collection and quality control

Factors potentially influencing the survival of nasopharyngeal carcinoma patients with distant metastasis were identified through a comprehensive literature review, including systematic reviews, original studies, and expert clinical opinions. At each center, blinded independent researchers systematically extracted detailed treatment information from medical records. The factors considered and recorded included: (1) sex; (2) age; (3) ECOG PS; (4) history of smoking and alcohol abuse; (5) serum/plasma EBV-DNA copy number; (6) serum lactate dehydrogenase (LDH) levels; (7) C-reactive protein (CRP) levels; (8) neutrophil, lymphocyte, platelet, and albumin levels; (9) Neutrophil-to-Lymphocyte Ratio (NLR), calculated as follows: neutrophil count (×109/L) / lymphocyte count (×109/L) [1]; (10) Platelet-to-Lymphocyte Ratio (PLR), calculated as follows: platelet count (×109/L) / lymphocyte count (×109/L) [2]; (11) Systemic Immune-Inflammation Index (SII), calculated as follows: platelet count (×109/L) × neutrophil count (×109/L) / lymphocyte count (×109/L) [3]; (12) Prognostic Nutritional Index (PNI), calculated as

follows: serum albumin (g/L) + 5 × total lymphocyte count (×109/L) [3]; (13) metastatic sites and lesions; (14) chemotherapy and immunotherapy regimen; (15) metastatic lesion response after CIT (Table S14).

A standardized multicenter database was set up to ensure data consistency and reliability. All data collectors received uniform training and used standardized forms for recording clinical information. After data collection, a random 20% sample of records from each center was independently reviewed by researchers and clinicians from other participating centers. A data accuracy rate of at least 90% was required, and any dataset not meeting this threshold had to be re-evaluated and corrected. Following this accuracy verification, data completeness was rigorously evaluated prior to tumor response assessment. Given that the proportion of missing data for essential covariates and survival endpoints was minimal (7.5%), patients with incomplete records were systematically excluded to strictly preclude imputation-induced bias. This ensured that all subsequent analyses were based on a complete-case dataset.

Supplementary Methods 3. Target trial emulation specifications

The 1.5-month grace period was strictly based on standard clinical workflows. In routine practice, the interval between post-CIT response evaluation and MDRT delivery typically spans two to six weeks to accommodate multidisciplinary consultation, radiotherapy simulation, target delineation, and plan quality assurance, making 1.5 months the shortest practical window that covers these steps while minimizing treatment strategy misclassification. Clones in the non-MDRT arm were artificially censored at the time of MDRT initiation, and clones in the MDRT arm at the end of the grace period if initiation had not occurred by then. Specifically, 296 clones (27.3%) in the non-MDRT arm and 778 clones (71.8%) in the MDRT arm were censored, which aligns with the real-world uptake of MDRT. Stabilized weights were not pursued because artificial censoring was a one-time, grace-period-confined event for each clone rather than a time-varying process accumulating over follow-up,

and the additional variance reduction would be limited. As detailed in Table S16, truncation at the 1st and 99th percentiles affected only 2% of the clones per arm without significantly altering the overall weight distribution, yielding weighted pseudo-populations that closely reproduced the original cohort size. This procedure compensated for censored individuals and maintained comparability between the two arms throughout the grace period**.**

Supplementary Methods 4. Sample size estimation

Considering the primary and secondary endpoints for this study, a strict hierarchical testing procedure was applied to strongly control the overall family-wise error rate at a two-sided significance level of 0.05. Specifically, PFS was tested first, and OS was subsequently evaluated at the 0.05 level only if the PFS benefit achieved statistical significance. No interim analysis was considered. The sample size for the ideal target trial was pre-estimated based on literature review and empirical baseline parameters using PASS software (version 15.0.5). We calculated that a hypothetical randomized trial would require 443 patients (111 for MDRT group, 332 for non-MDRT group), targeting 348 progression events, to achieve 80% power (two-sided $\alpha = 0.05$) for a PFS HR of 0.70 (median 23.0 versus 16.0 months) over a 24-month accrual and an additional 36-month follow-up, with a 5% annual loss to follow-up [4, 5]. In our TTE, we exceeded this minimum to include as many patients as possible, aiming to improve generalizability and enhance statistical power.

Supplementary Methods 5. Sensitivity analyses

Multiple analyses were conducted to support the robustness of the primary results. First, a conditional landmark analysis at 1.5 months after T0 was performed to further mitigate potential immortal time bias. This 1.5-month window corresponded to the grace period allowed for initiating MDRT, ensuring balanced follow-up and reducing bias related to delayed treatment initiation. Within this landmark cohort, stabilized inverse probability of treatment weighting (sIPTW) was utilized to emulate randomization and address the imbalance of potential confounders. A directed acyclic

graph was created to identify these confounders (Figure S14), comprising ECOG performance status, gender, age, metastatic sites (liver, bone, lung, and other), number of metastatic lesions, metastatic lesion response to CIT, cycles of first-line CIT, and post-CIT levels of EBV DNA, CRP, LDH, SII, and PNI. Variables exhibiting a standardized mean difference (SMD) of less than 0.1 were deemed balanced. Second, the TTE procedures were repeated using alternative grace periods of 1.0 and 2.0 months. Third, an analysis without artificial censoring was performed during the follow-up. Patients surviving beyond the 1.5-month landmark were classified by actual treatment received, with sIPTW applied to balance covariates. Fourth, an unadjusted analysis of the landmark population was performed using unweighted Cox models. Fifth, propensity score matching (PSM) was conducted using a 2:1 nearest-neighbor algorithm without replacement and a caliper width of 0.2 standard deviations of the logit propensity score. Finally, the E-value was calculated to evaluate the potential impact of unmeasured confounding on the observed association between treatment and survival outcomes.

Supplementary Methods 6. Feature selection and model development details

Variance inflation factors were calculated to verify that multicollinearity was fully resolved among the retained features (Figure S15), and the overlap of variables selected by the four algorithms is presented in a Venn diagram. Global hyperparameter optimization was conducted prior to evaluation to maximize predictive performance and minimize overfitting, followed by 10-fold cross-validation within the training set. The mathematical formulation for the CB score is presented in the following Eq. (1)

$$\text{CB score} = \alpha \times iAUC_{0\rightarrow3yr} + \alpha \times (1 - IBS_{0\rightarrow3yr}) \quad (1)$$

$$\alpha = 0.5 \quad (2)$$

where $iAUC_{0\rightarrow3yr}$ and $IBS_{0\rightarrow3yr}$ represent the arithmetic means of the iAUC and the IBS derived from the 10-fold internal cross-validation, respectively. Specifically, the iAUC represents the weighted integration of time-dependent receiver operating characteristic (ROC) curves spanning a clinical interval up to 3 years, while the IBS

calculates the integral of the overall probability calibration error across the same period. By setting the weight factor to 0.5, equal emphasis is placed on discrimination and calibration in the CB_{score}. The term (1- $IBS_{0\rightarrow 3yr}$) is used to align with the positive direction of the iAUC.

Supplementary Methods 7. Causal inference implementation details

The baseline prognostic score was fitted using only the censoring weights of the non-MDRT arm. These weights are functions of baseline covariates and restore the representation of clones that were artificially censored upon deviation from the non-MDRT strategy. Neither outcomes nor weights from the MDRT arm entered this calculation, ensuring that the score represents the pure counterfactual prognosis had no patient received consolidative MDRT. Of the seven predictors retained during feature screening, only six entered this score. MDRT was excluded by construction and was used solely as a predictor in the prognostic models of observed survival described previously. Both arm-specific T-learner models used identical hyperparameters, and the number of boosting iterations was selected separately within each arm by fivefold cross-validation. Owing to the cloning step, each model was trained on the full set of clones assigned to the corresponding strategy rather than only on patients who actually received each treatment. Effective sample sizes and weighted event counts are provided in Table S17. For covariate adjustment in the G-computation step, no additional covariates beyond the six predictors intrinsically selected by the optimal models were entered. These six predictors were pre- and post-CIT EBV DNA, pre-CIT LDH, post-CIT CRP, number of metastatic lesions, and tumor response. The two arm-specific models were used to predict individual conditional survival curves under both the MDRT and non-MDRT strategies from 0 to 3 years. Within each RPA-defined risk group, these curves were averaged with equal weight over the empirical covariate distribution of that group, yielding the marginal expected survival trajectory as if the entire group had been uniformly treated or untreated. This stratified standardization was performed independently in the derivation and geographical external validation cohorts.

Supplementary Figures

A

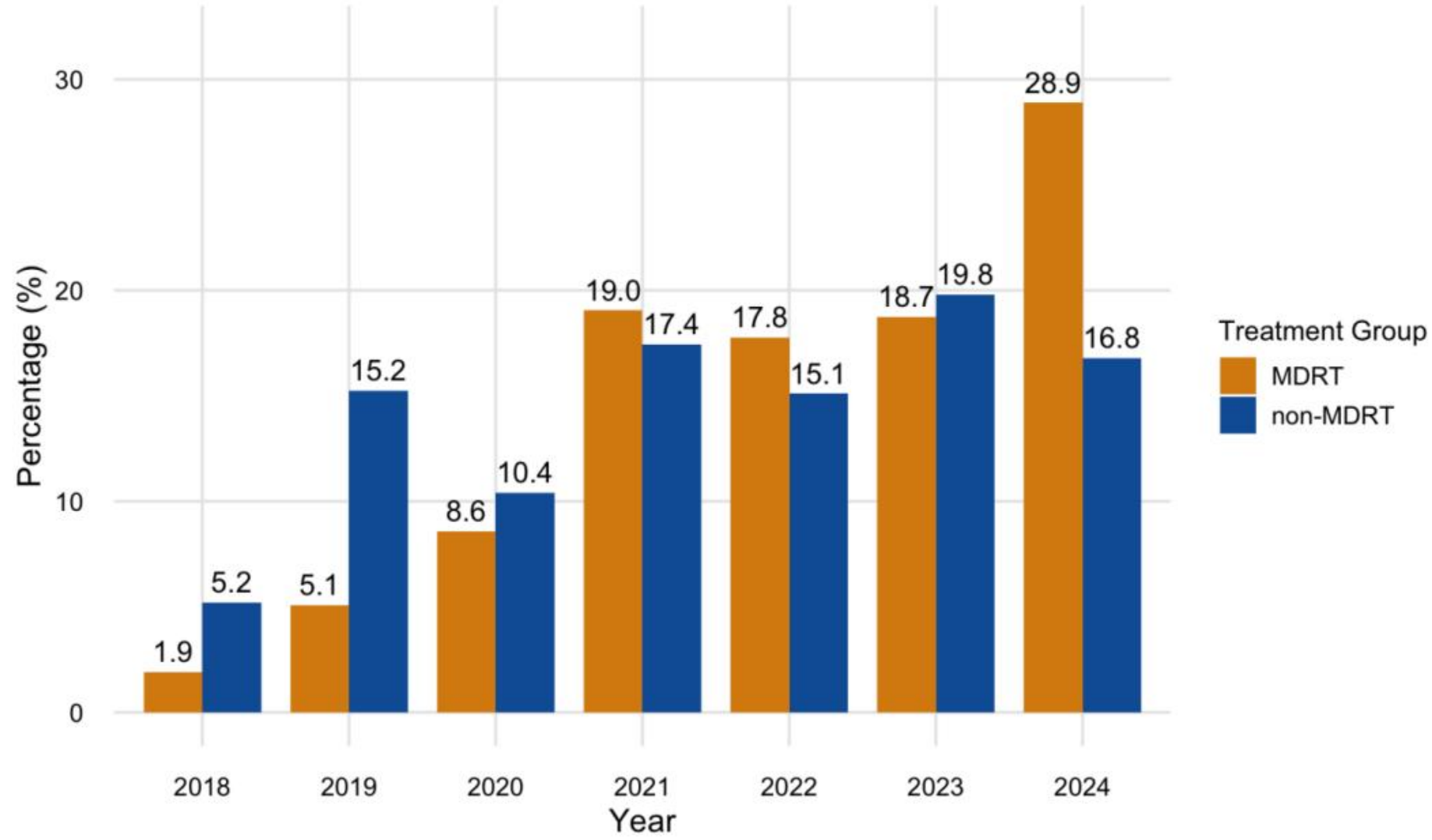

B

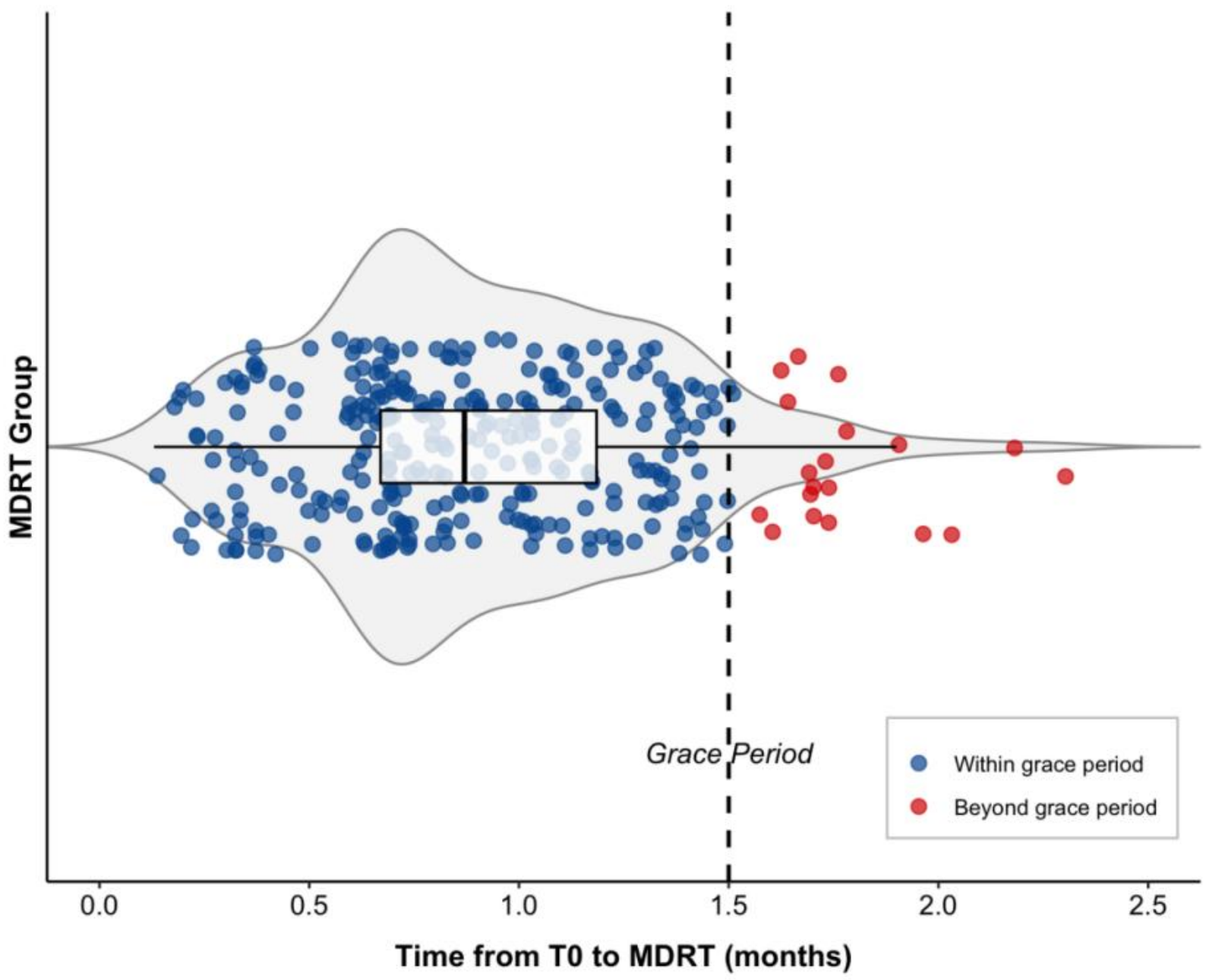

Figure S1. Annual distribution of patients by treatment group and density and distribution of time from tumor response evaluation (T0) to the initiation of MDRT. A. Annual distribution of patients by treatment group. B. Density and distribution of time from tumor response evaluation (T0) to the initiation of MDRT. Bar chart showing the annual proportions of patients in the MDRT and non-MDRT groups from 2018 to 2024. The figure illustrates a gradual increase in the use of MDRT over time, reflecting its expanding clinical adoption in real-world practice.

Abbreviation: MDRT, metastasis-directed radiotherapy.

A

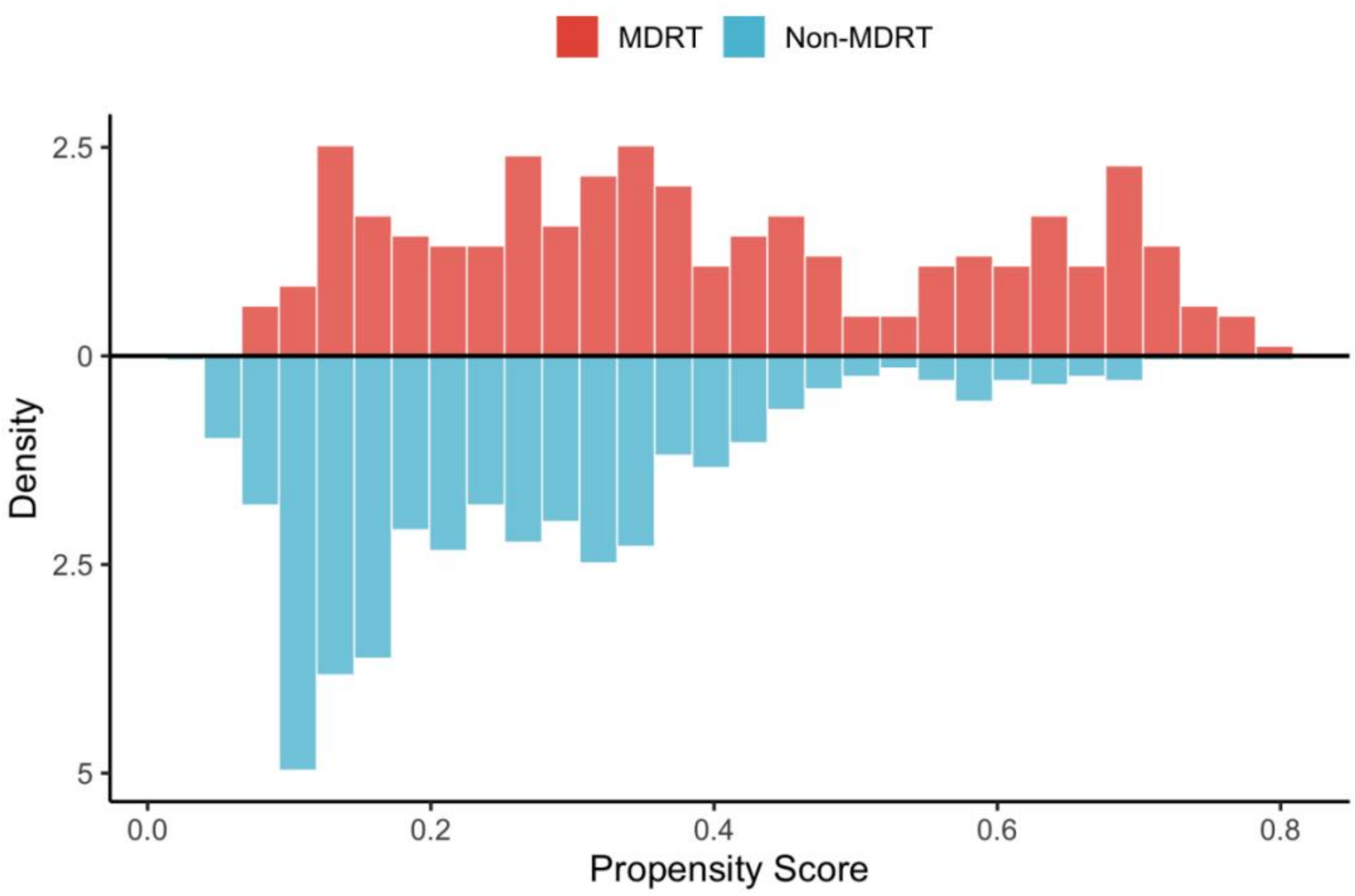

B

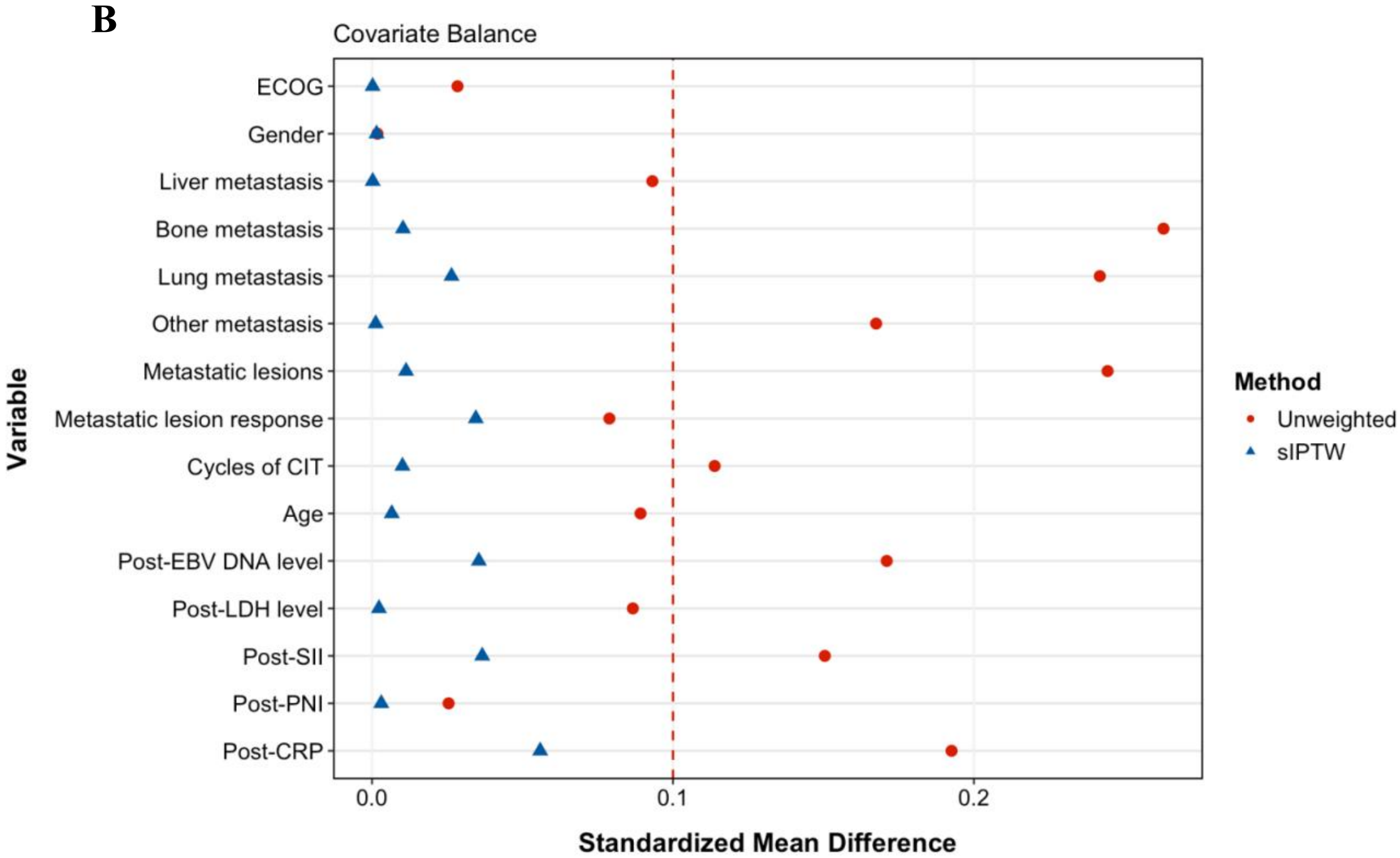

Figure S2. The distribution of propensity scores and standardized mean differences after applying sIPTW adjustments. A. The distribution of propensity scores after applying sIPTW adjustments. B. Standardized mean differences before and after applying sIPTW.

Abbreviation: post-, post-chemoimmunotherapy; ECOG, Eastern Cooperative Oncology Group; EBV, Epstein-Barr virus; LDH, lactate dehydrogenase; CRP, C-reactive protein; SII, systemic immune-inflammation index; PNI, prognostic nutritional index; CIT, chemoimmunotherapy; sIPTW, stabilized inverse probability of treatment weighting; MDRT, metastasis-directed radiotherapy.

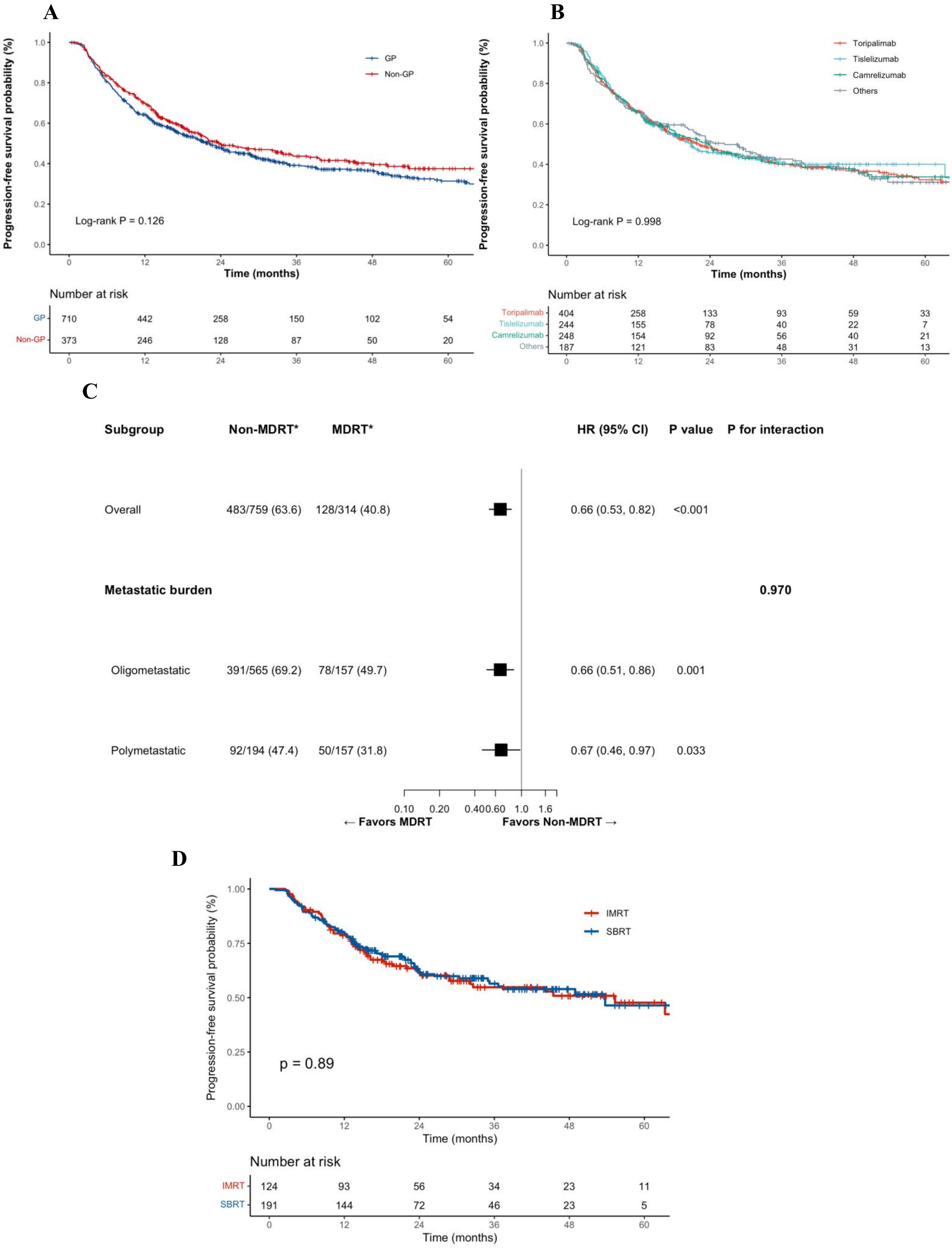

Figure S3. Kaplan-Meier estimates of progression-free survival stratified by systemic treatment regimens and radiotherapy modalities and subgroup forest plot stratified by metastatic burden. A. PFS comparison between patients receiving the GP (gemcitabine plus platinum) regimen versus non-GP regimens. B. PFS comparison across the predominant immunotherapy agents. C. Impact of radiotherapy modalities on survival outcome in the MDRT group. D. Forest plot of progression-free survival stratified by metastatic burden in the sIPTW-adjusted cohort.

*The total number of patients in the two groups varies slightly in the post-sIPTW pseudo-data set due to weight truncation.

Abbreviation: IMRT, intensity-modulated radiation therapy; SBRT, stereotactic body radiation therapy.

A

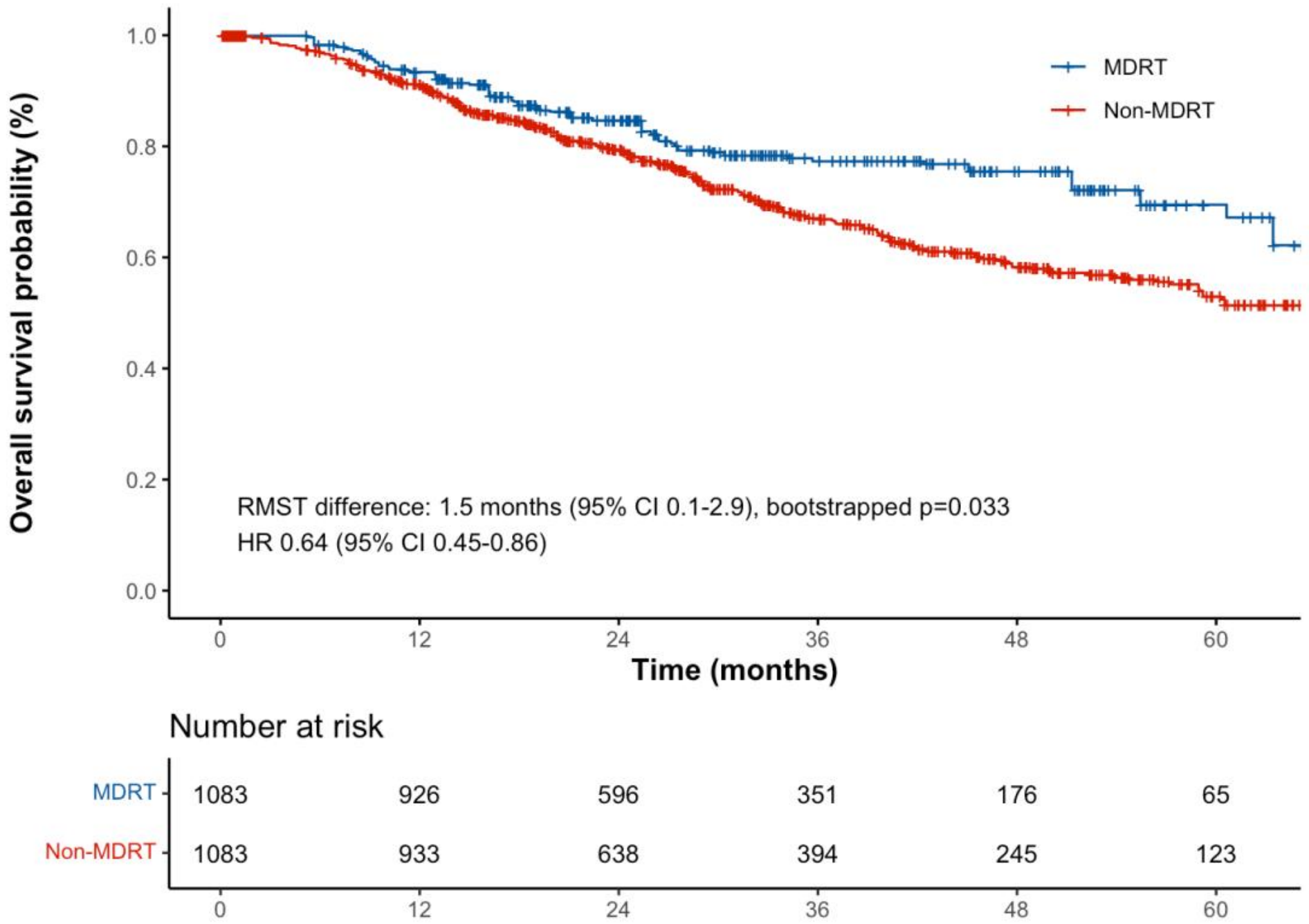

B

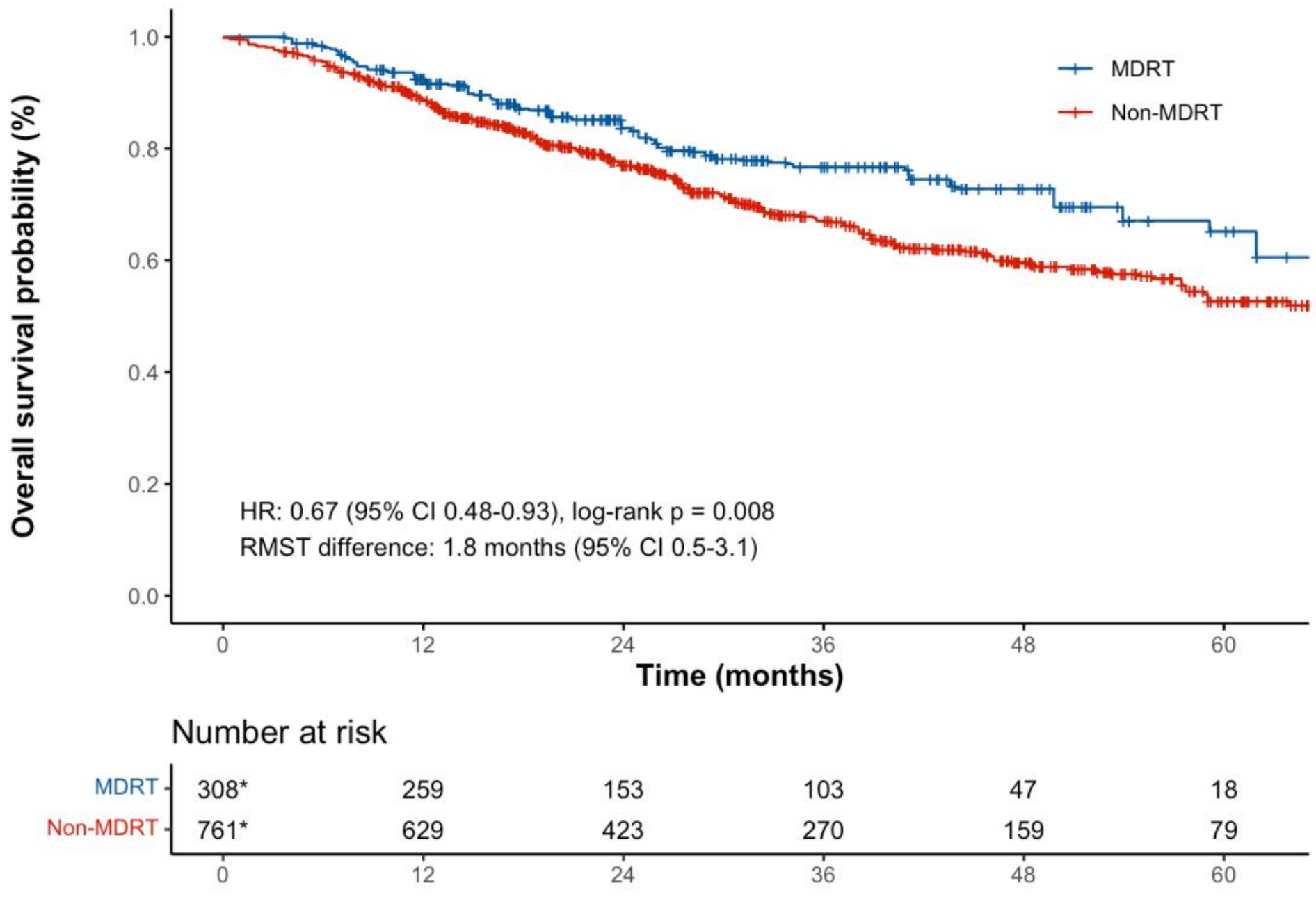

Figure S4. Kaplan–Meier curves of overall survival after cloning-censoring-weighting and sIPTW analyses.

Abbreviation: sIPTW, stabilized inverse probability of treatment weighting; MDRT, metastasis-directed radiotherapy.

*The total number of patients in the two groups varies slightly in the post-sIPTW pseudo-data set due to weight truncation.

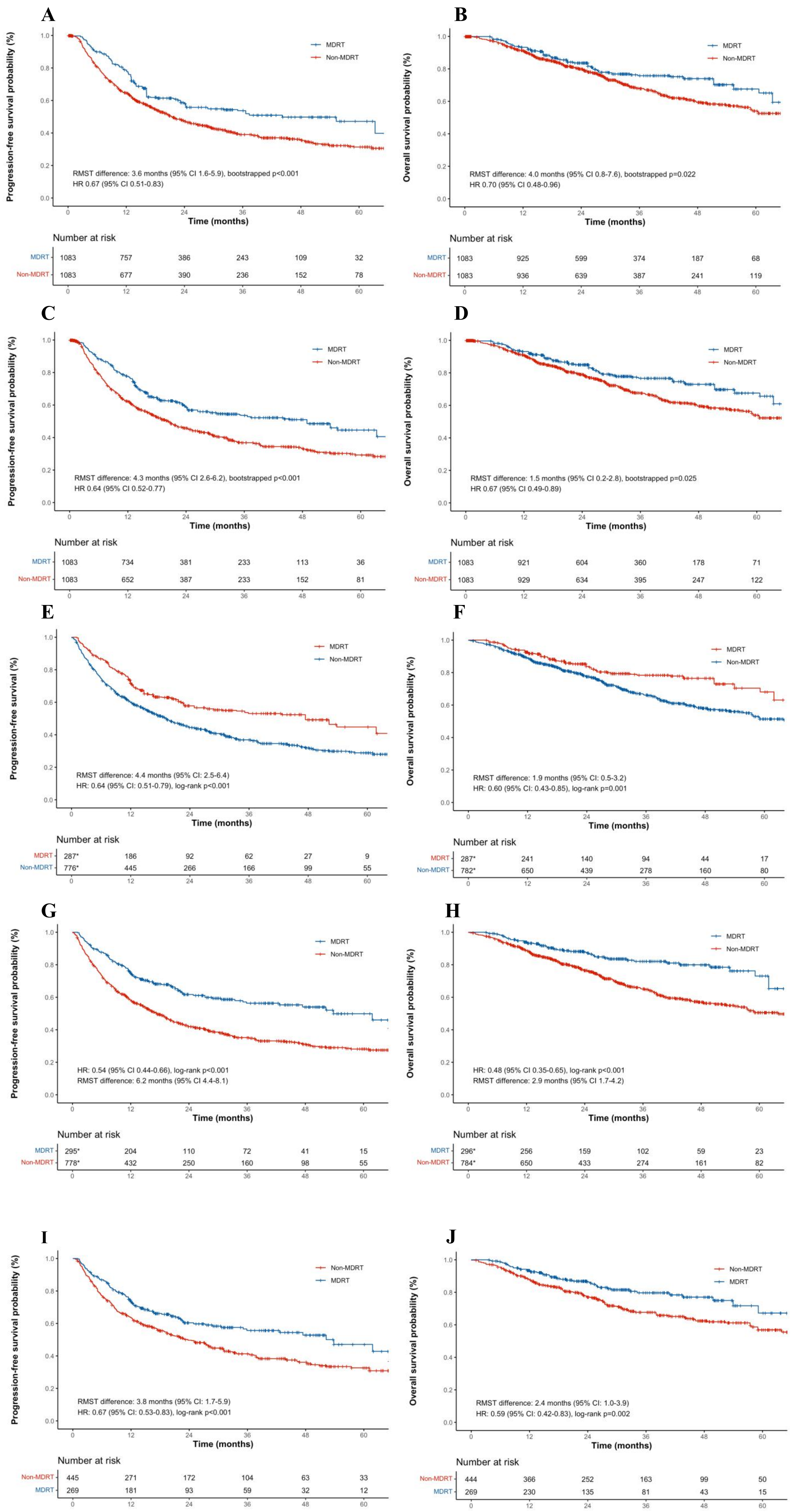

Figure S5. Kaplan-Meier curves of progression-free survival and overall survival across sensitivity analyses. Kaplan-Meier curves of progression-free survival (A) and overall survival (B) after cloning-censoring-weighting analyses with a 1 month grace period. Kaplan-Meier curves of progression-free survival (C) and overall survival (D) after

cloning-censoring-weighting analyses with a 2 months grace period. Kaplan-Meier curves of progression-free survival (E) and overall survival (F) in the target trial emulation without artificial censoring. Kaplan-Meier curves of progression-free survival (G) and overall survival (H) in unadjusted population. Kaplan-Meier curves of progression-free survival (I) and overall survival (J) in propensity score matching analysis. The propensity score matching analysis was performed within the 1.5-month landmark population using a 2:1 nearest-neighbor method without replacement and a caliper width of 0.2 standard deviations of the logit of the propensity score. The propensity scores were calculated using a logistic regression model, factoring in predefined covariates including age, sex, ECOG, metastatic sites, number of metastatic lesions, tumor response, cycles of CIT, and post-biomarkers (EBV DNA, LDH, SII, PNI, and CRP).

* in (E) and (F): The total number of patients in the two groups varies slightly in the post-sIPTW pseudo-data set due to conditional landmark exclusions and weight truncation.

* in (G) and (H): The total number of patients in the two groups varies slightly due to conditional landmark exclusions.

Abbreviation: MDRT, metastasis-directed radiotherapy.

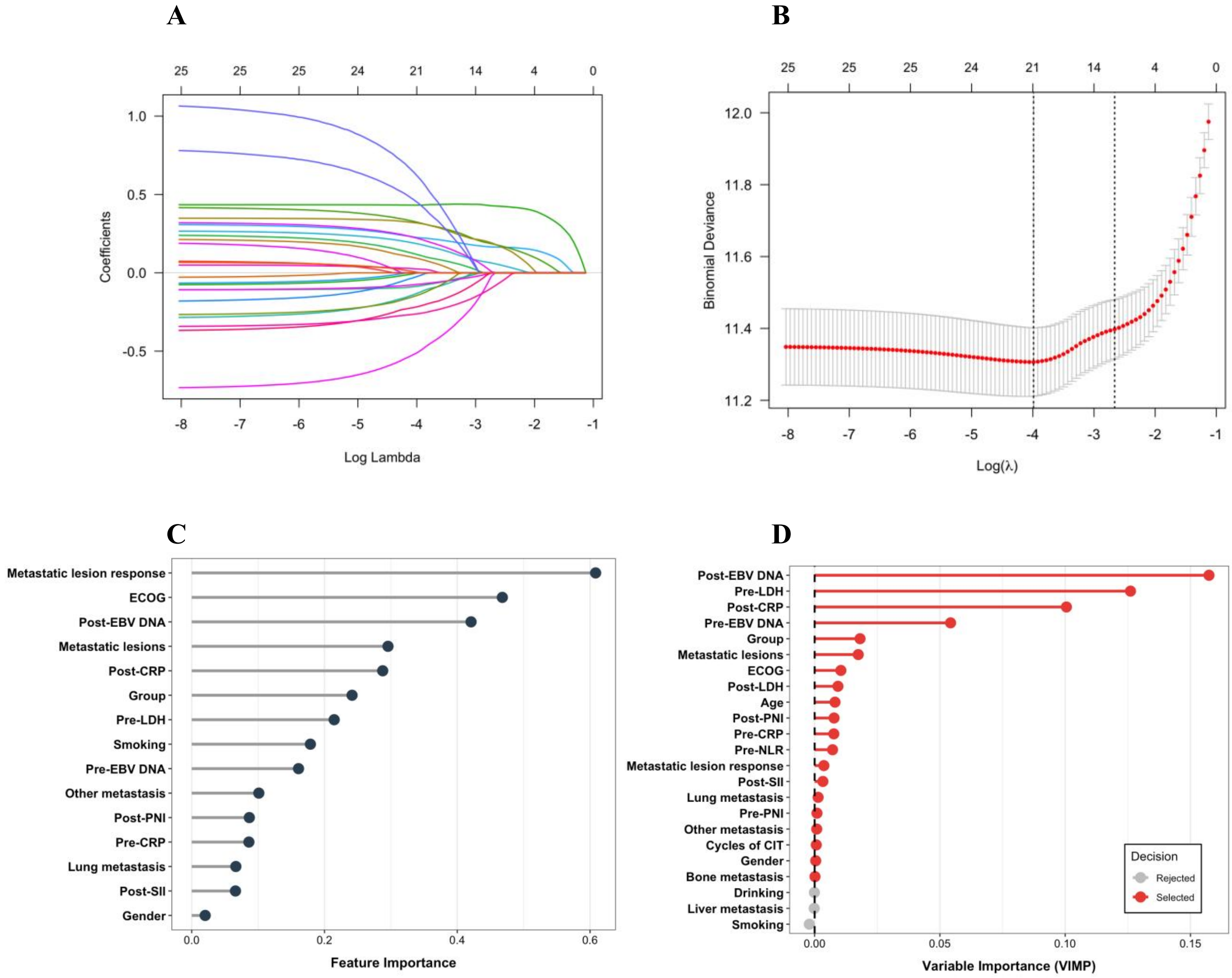

Figure S6. Variable selection using Least Absolute Shrinkage and Selection Operator, Random Survival Forest, and Elastic Net. A. Cross-validation error plot for LASSO. B. Coefficient path plot for LASSO. The optimal λ (λ_{min}) determined via 10-fold cross-validation. C. Feature importance plot derived from the Group Elastic Net model. D. Variable Importance (VIMP) plot generated by the Random Survival Forest (RSF) model.

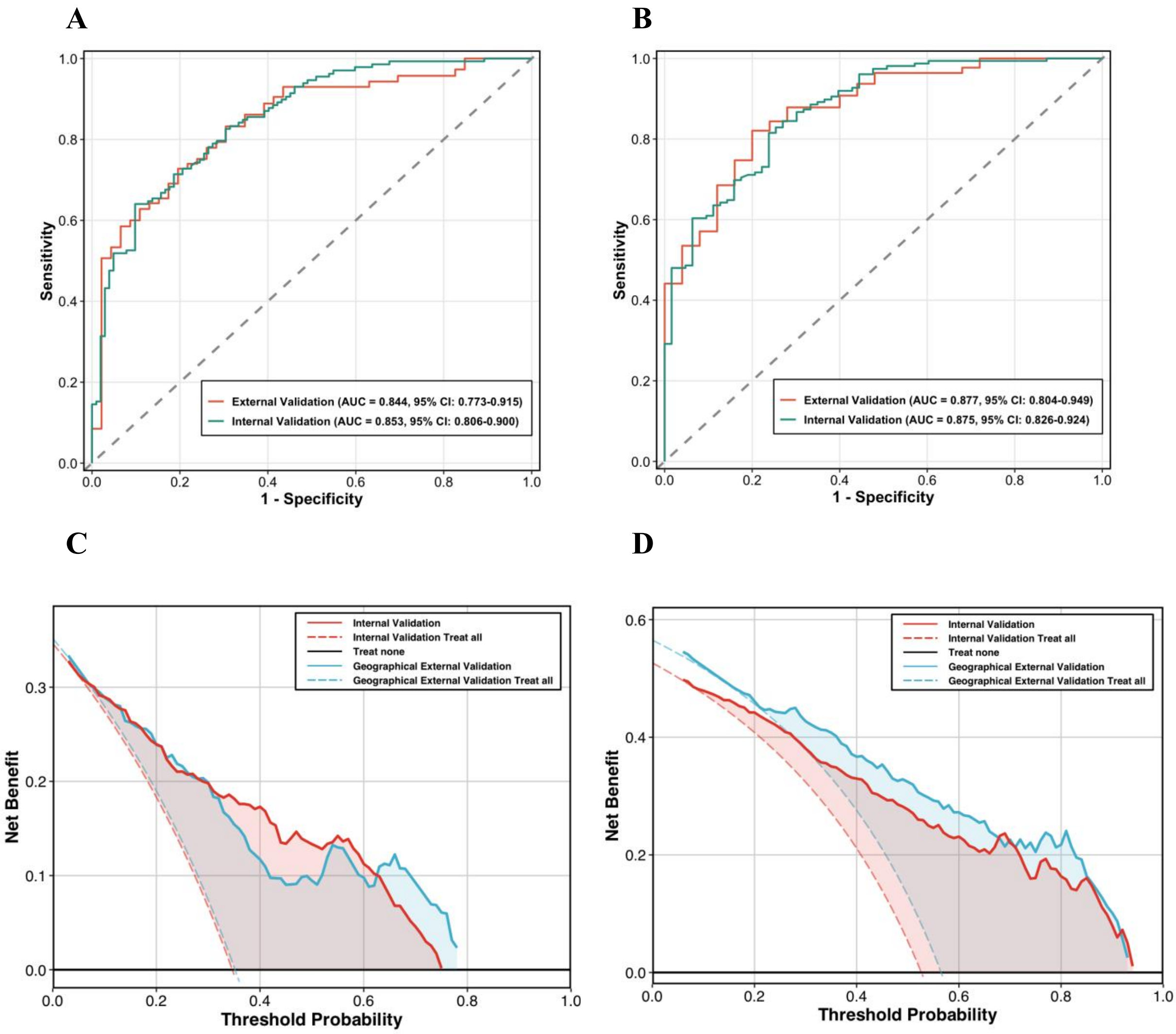

Figure S7. Model performance evaluation in the internal validation cohort and geographical external validation cohort. A. ROC curves for predicting 1-year PFS in the internal validation cohort and geographical external validation cohort. B. ROC curves for predicting 2-year PFS in the internal validation cohort and geographical external validation cohort. C. Decision curve analysis for predicting 1-year PFS in the internal validation cohort and geographical external validation cohort. D. Decision curve analysis for predicting 2-year PFS in the internal validation cohort and geographical external validation cohort.

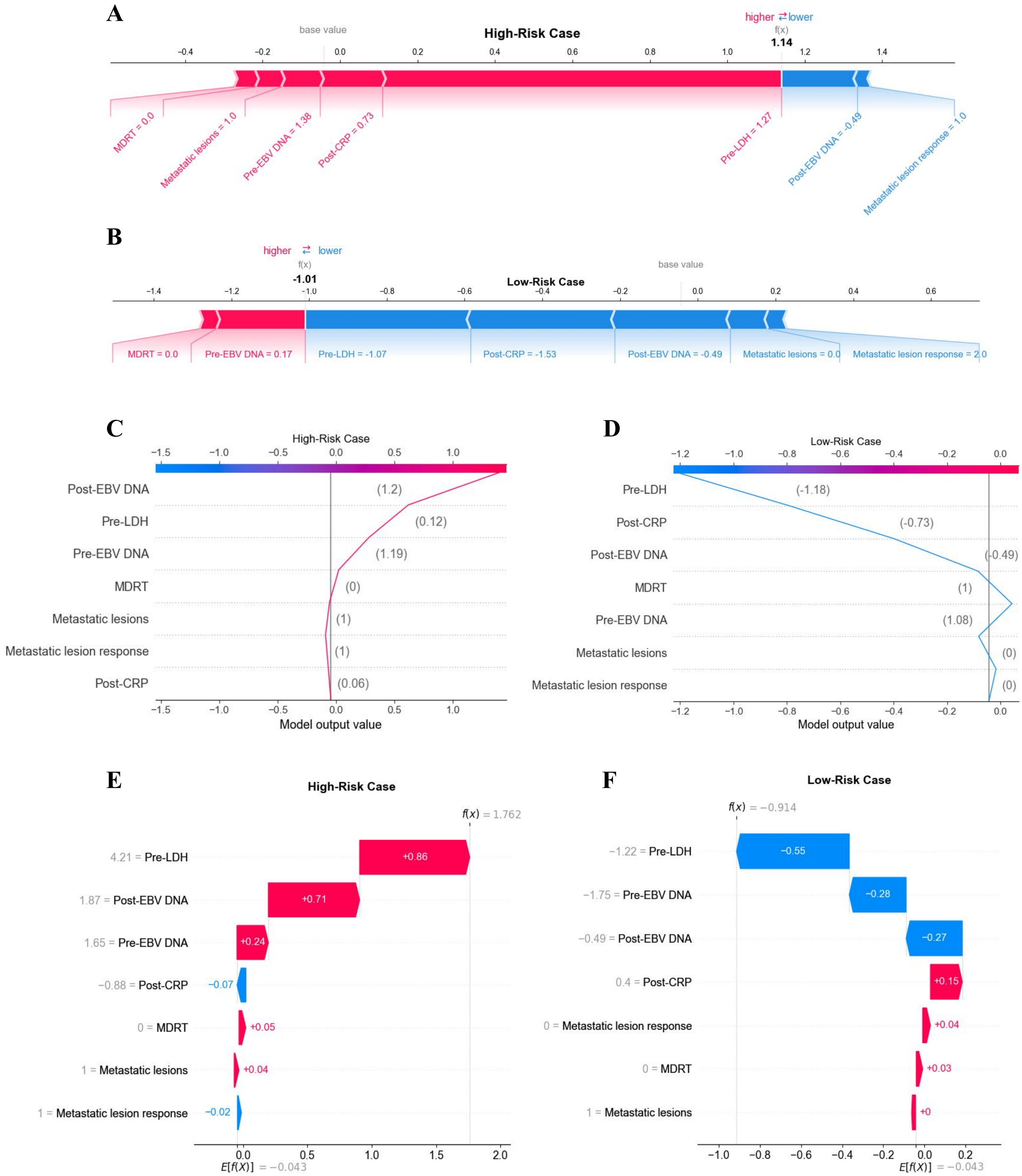

Figure S8. Local SHAP explanations for representative samples. SHAP force plots showing the feature contributions to model prediction in a high risk case (A) and a low risk case (B). SHAP decision plots illustrating how each feature contributes to the model's prediction in a high risk case (C) and a low risk case (D). SHAP waterfall plots illustrating the cumulative effect of each variable on the final prediction in a high risk case (E) and a low risk case (F).

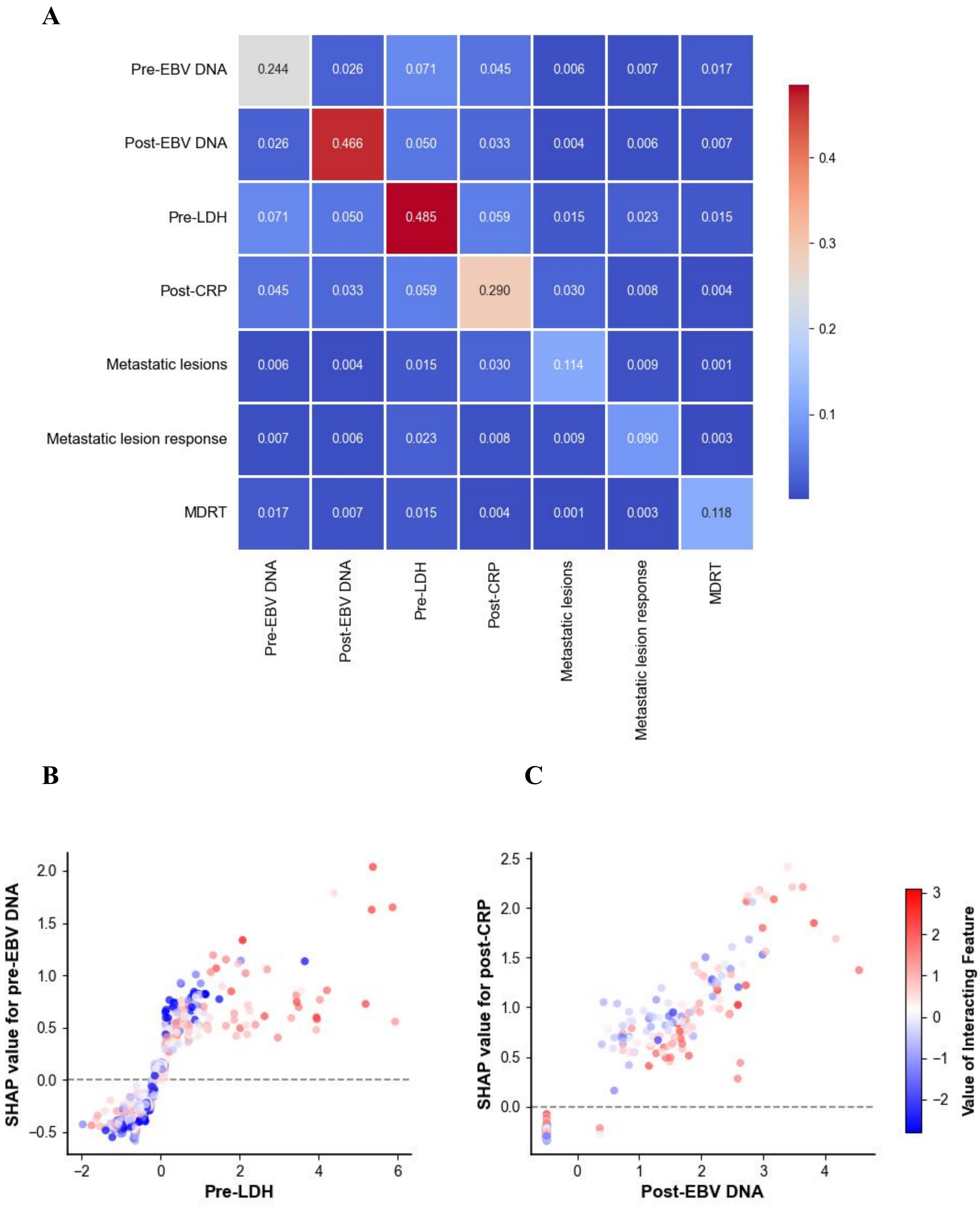

Figure S9. SHAP interaction heatmap and SHAP dependence plots of key predictive features. A. SHAP interaction heatmap. This plot quantifies pairwise feature interactions, where darker colors represent stronger interaction effects. B. pre-LDH. C. post-EBV DNA.

A

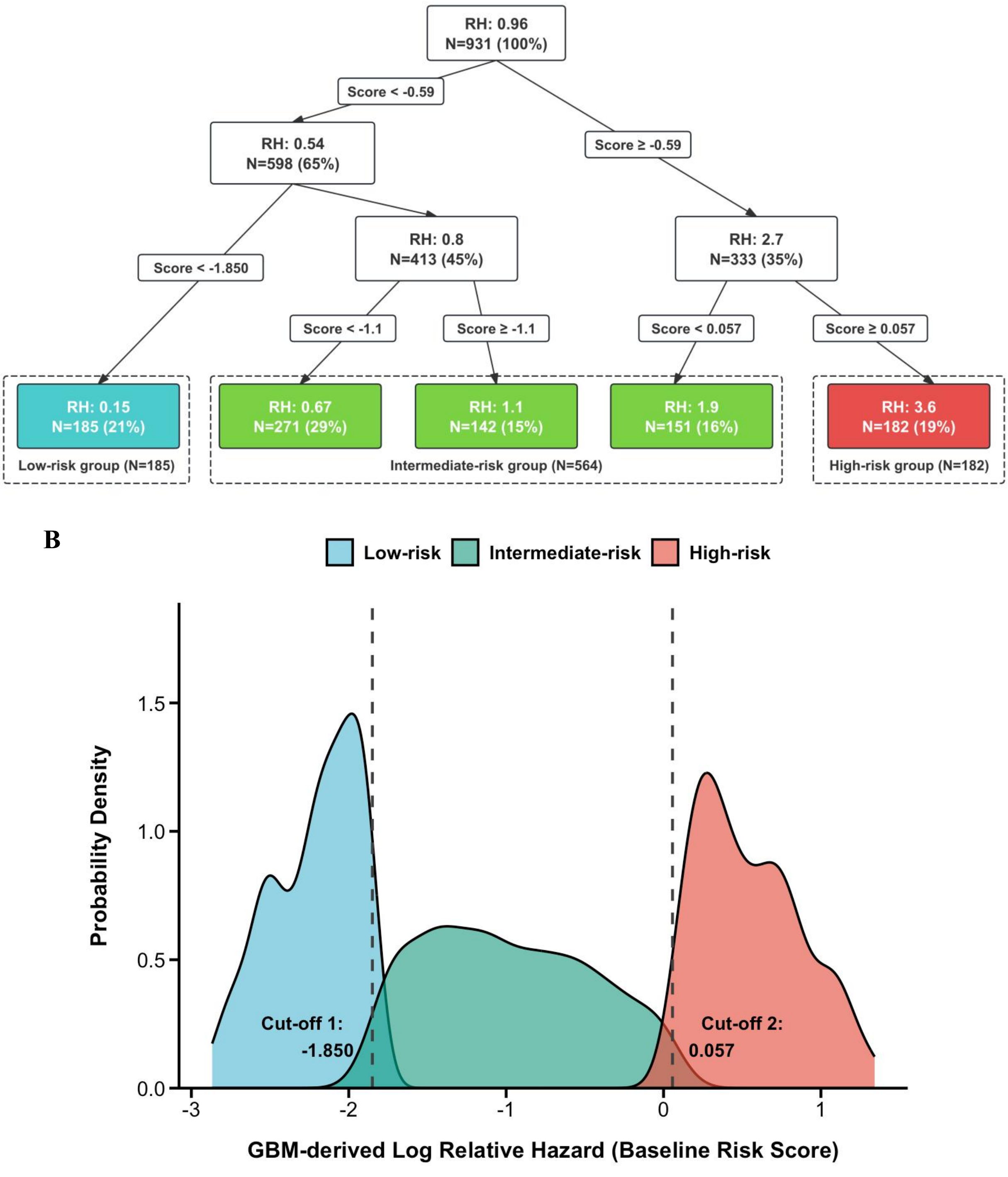

B

Figure S10. Identification of prognostic risk groups using recursive partitioning analysis. A. The pruned RPA tree stratified patients into distinct prognostic risk subgroups based on the baseline risk score derived from the natural history pseudo-cohort. The values within each node represent the RH of progression-free survival events compared to the baseline hazard.

B. Density distribution of the GBM-derived baseline risk score.

Abbreviation: RH, relative hazard.

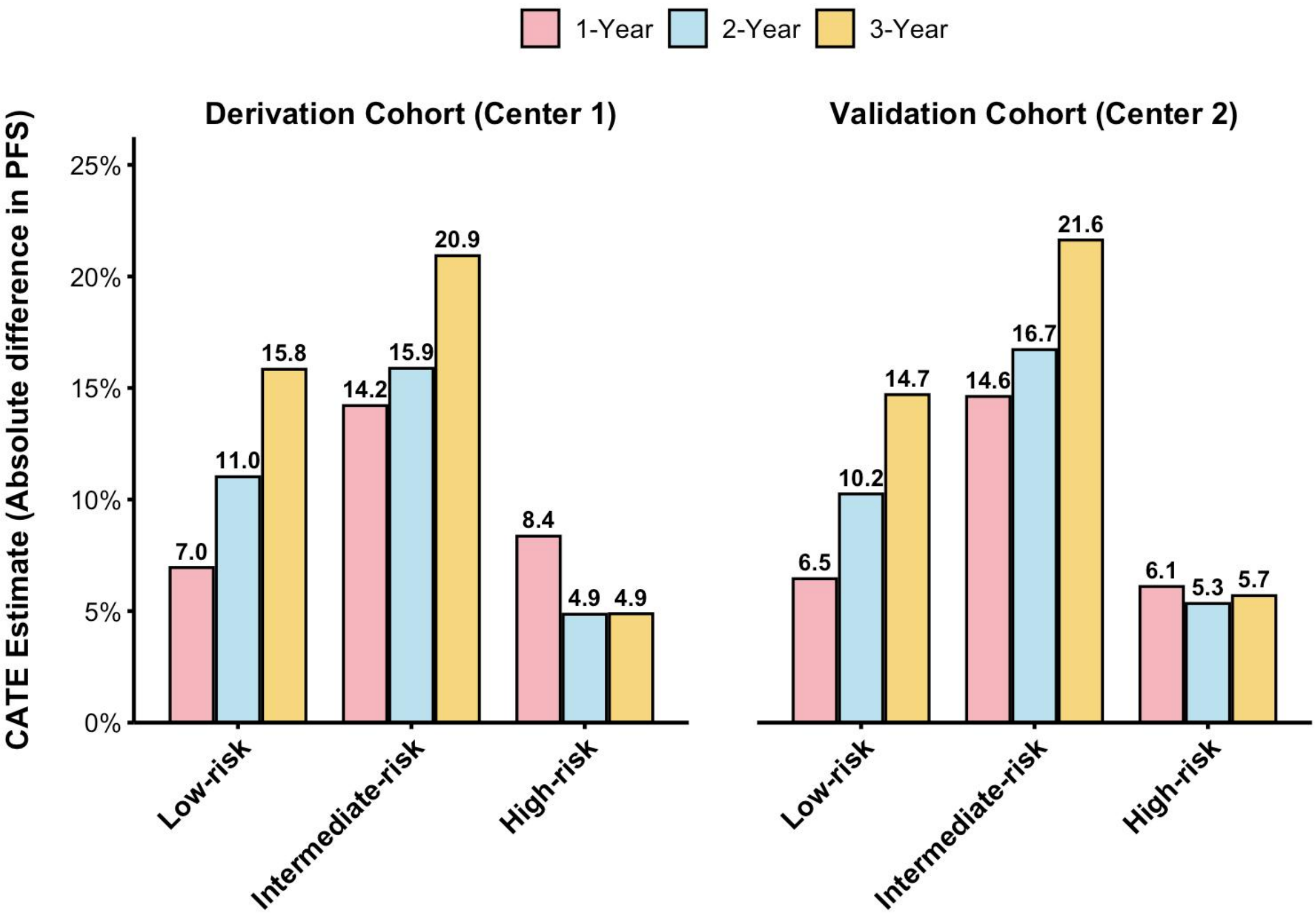

Figure S11. Time-varying CATE of MDRT across prognostic risk groups.

Abbreviation: CATE, conditional average treatment effects; MDRT, metastasis-directed radiotherapy.

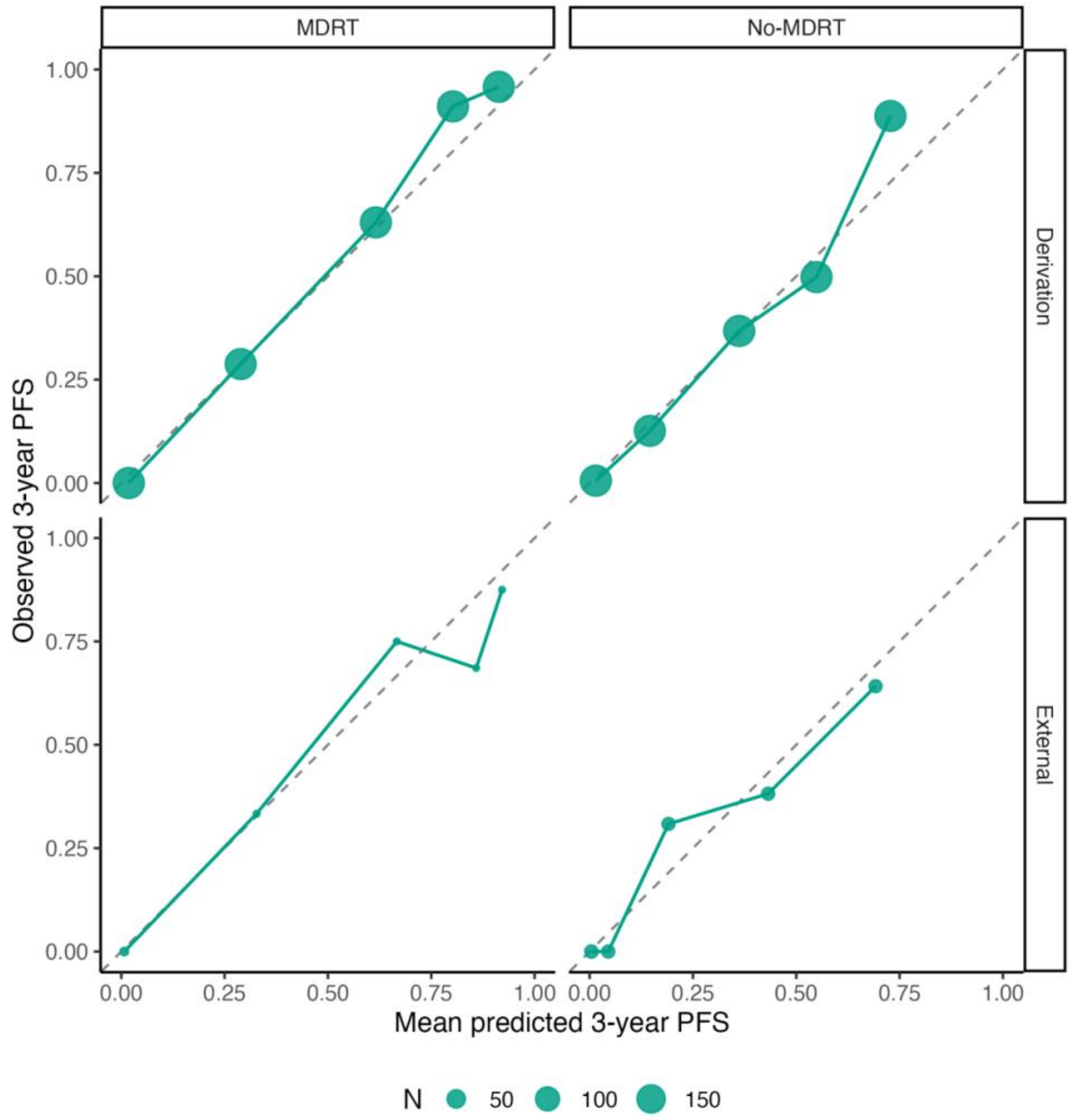

Figure S12. Calibration curves of the arm-specific T-learner models for predicting 3-year progression-free survival. The plots illustrate the agreement between the mean predicted 3-year PFS and the observed 3-year PFS within the MDRT and non-MDRT arms. Performance is evaluated separately in the derivation cohort (top row) and the geographical external validation cohort (bottom row). Dot sizes are proportional to the number of patients in each predicted probability quintile.

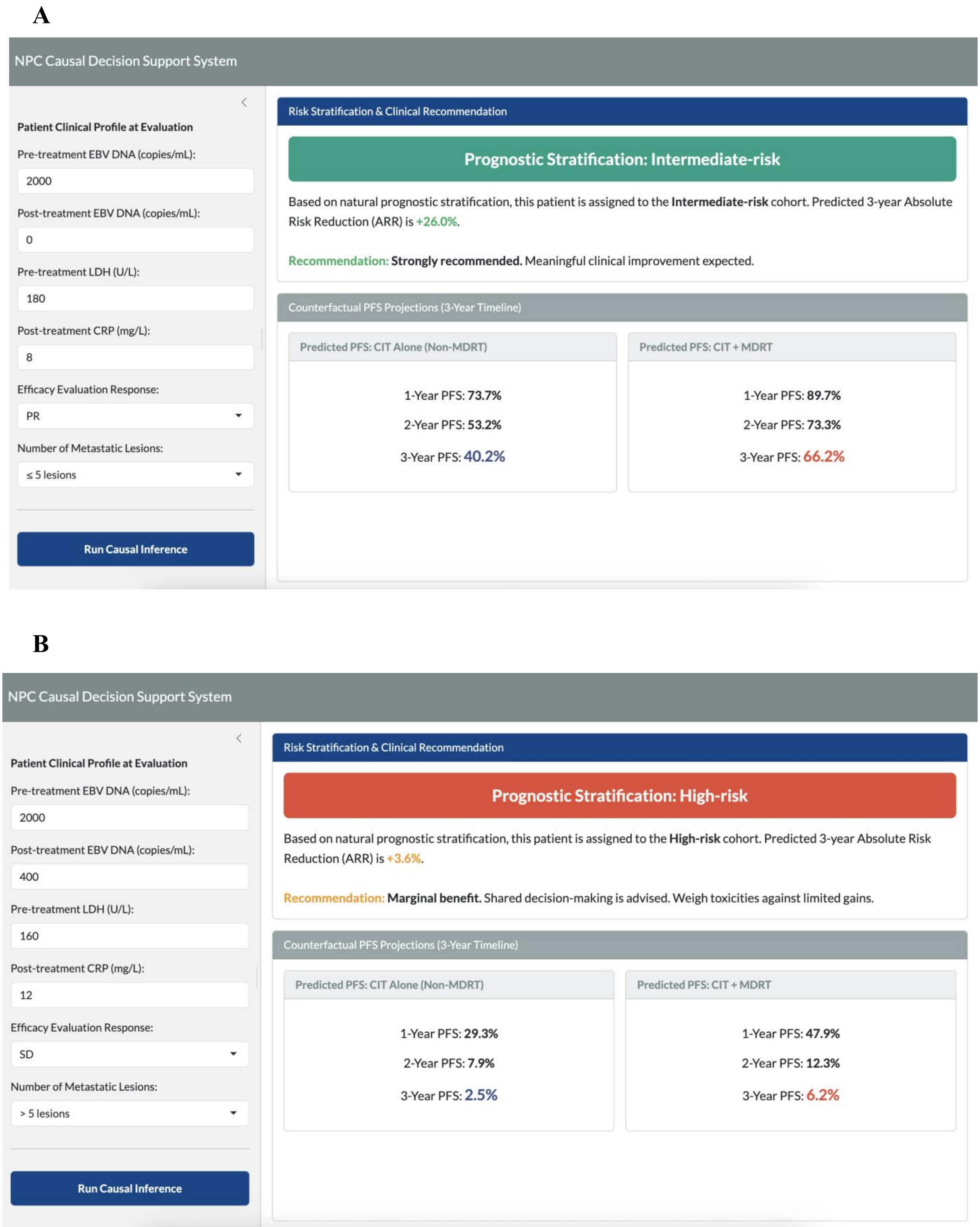

Figure S13. Web-based clinical decision-support platform for individualized progression-free survival prediction and radiotherapeutic benefit estimation in metastatic nasopharyngeal carcinoma.

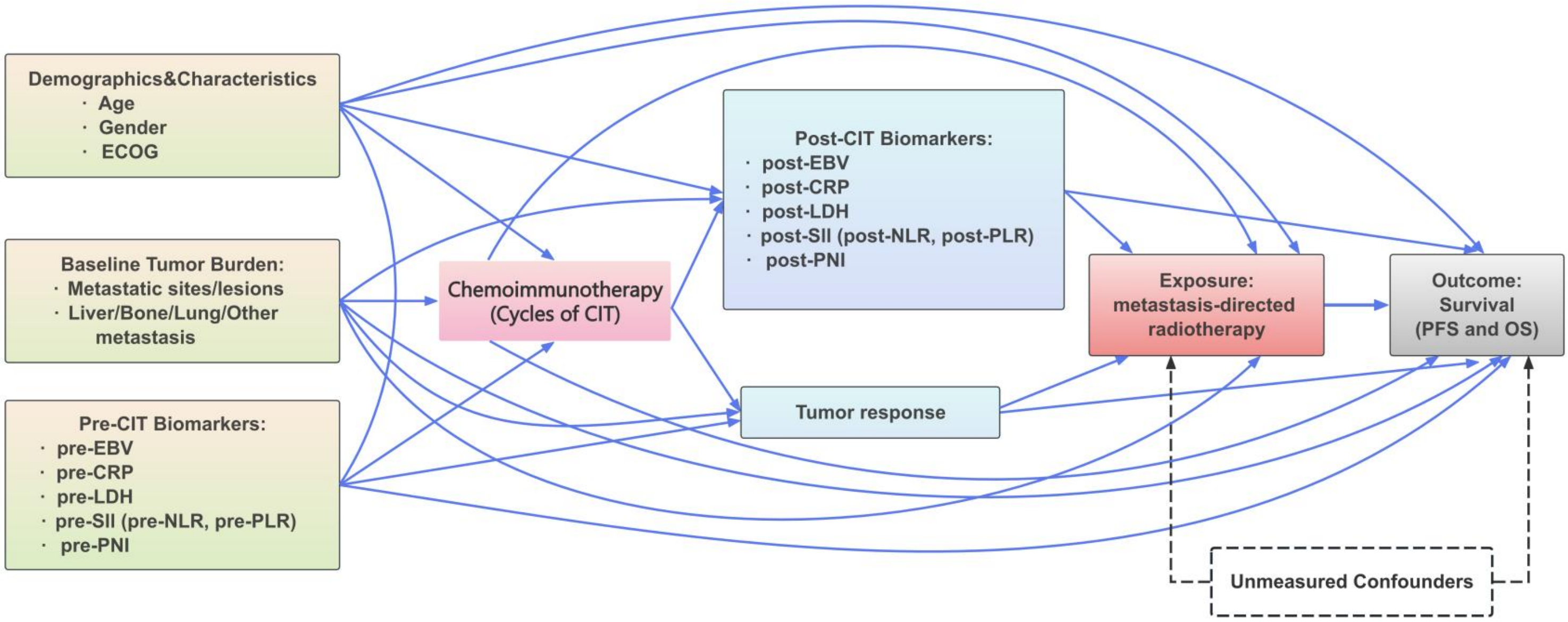

Figure S14. Directed acyclic graphs used to identify confounding covariates for the survival adjustment sets in this study.

Abbreviation: Pre-, pre-chemoimmunotherapy; Post-, post-chemoimmunotherapy; ECOG PS, Eastern Cooperative Oncology Group performance status; EBV, Epstein-Barr virus; LDH, lactate dehydrogenase; CRP, C-reactive protein; NLR, neutrophil-to-lymphocyte ratio; PLR, platelet-to-lymphocyte ratio; SII, systemic immune-inflammation index; PNI, prognostic nutritional index; CR, complete response; PR, partial response; SD, stable disease; CIT, chemoimmunotherapy.

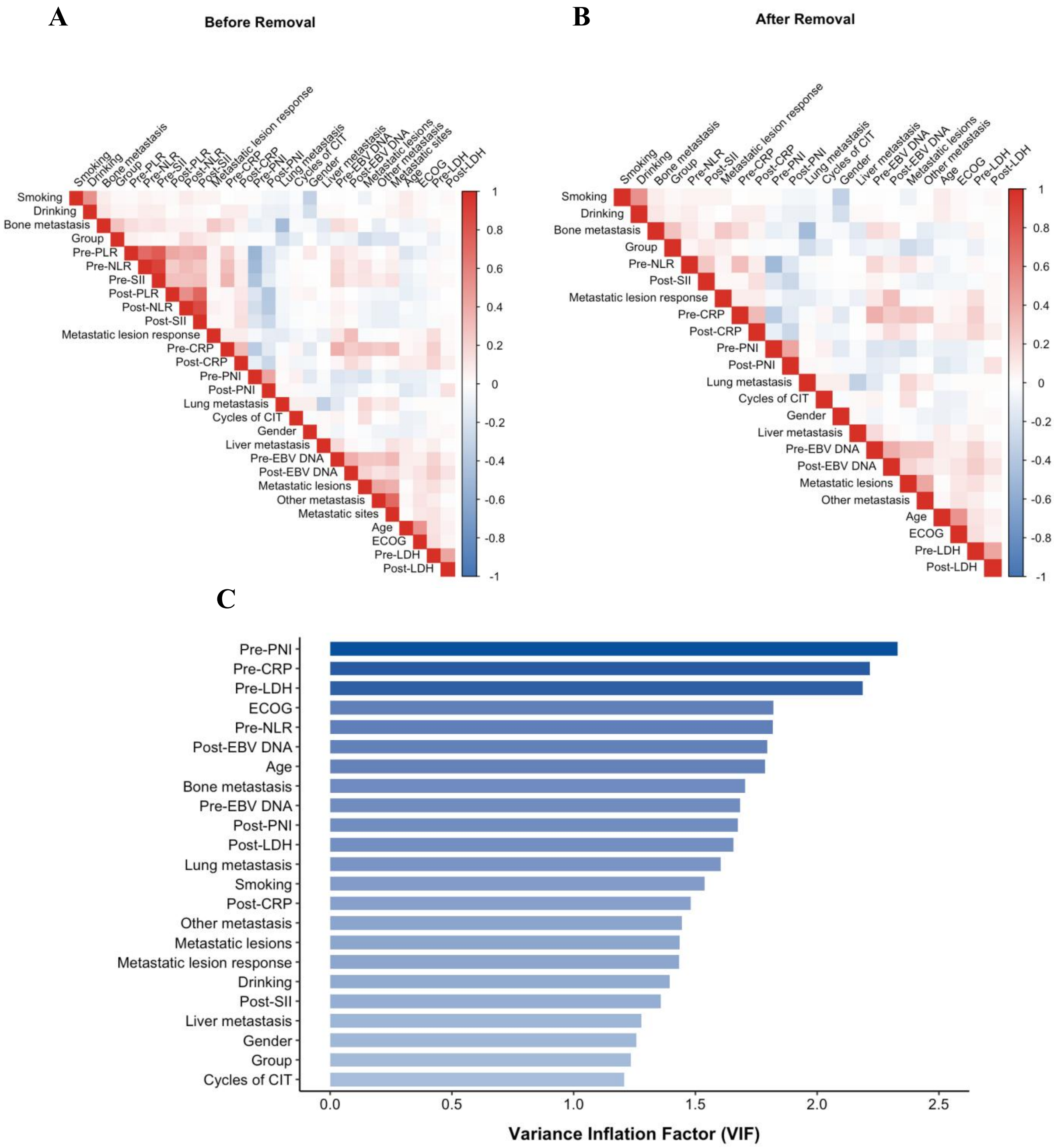

Figure S15. Correlation filtering of highly correlated variables. A. Heatmap of correlation analyses among variables before correlation filtering. B. Heatmap of correlation analyses among variables after correlation filtering. C. Variance inflation factor (VIF) for the remaining variables.

Abbreviation: pre-, pre-chemoimmunotherapy; post-, post-chemoimmunotherapy; ECOG PS, Eastern Cooperative Oncology Group performance status; EBV, Epstein-Barr virus; LDH, lactate dehydrogenase; CRP, C-reactive protein; NLR, Neutrophil-to-Lymphocyte Ratio; PLR, Platelet-to-Lymphocyte Ratio; SII, Systemic Immune-Inflammation Index; PNI, Prognostic Nutritional Index; CR, complete response; PR, partial response; SD, stable disease; CIT, chemoimmunotherapy; MDRT, metastasis-directed radiotherapy.

The filtering process involves the following steps:

(1) Calculate the correlation coefficient matrix of the variables.

(2) Identify the pair of variables with the highest absolute correlation coefficient (denoted as variables A and B).

(3) Calculate the correlation coefficients of A and B with all other predictor variables separately.

(4) If the average correlation coefficient of A is greater, remove variable A; otherwise, remove variable B.

(5) Repeat steps 2-4 until the absolute value of all correlation coefficients falls below 0.6.

The correlation coefficients are calculated as follows:

(1) For continuous and ordered categorical variables, use Spearman's Rank Correlation Coefficient.

$$\rho = 1 - \frac{6\sum d_i^2}{n(n^2-1)}$$

where ρ is Spearman's rank correlation coefficient; d_i is the difference between the ranks of each pair of observation; n is the number of observations.

(2) For unordered categorical variables, apply Theil's U,

$$H(X) = -\sum_{i=1}^{n} p(x_i)\log(p(x_i))$$

$$H(X|Y) = -\sum_{y \in Y}\sum_{x \in X} p(x,y)\log(p(x|y))$$

$$U = \frac{H(X) - H(X|Y)}{H(X)}$$

where $H(X)$ is the entropy of variable X ; p (x_i) is the probability of category x_i; p(x,y) is the joint probability of category x and category y occurring together; n is the total number of categories; U is the Theil's U; $H(X|Y)$ is the conditional entropy of X given Y.

(3) For correlations involving unordered categorical variables and continuous or ordered categorical variables, employ Correlation Ratios.

$$\eta^2 = \frac{SS(Betweengroups)}{SS(Total)}$$

where $SS(Betweengroups)$ is the sum of squares for the effect of the independent variable and $SS(Total)$ is the total sum of squares.

Supplementary Tables

Table S1. Patient baseline characteristics before and after applying stabilized inverse probability of treatment weighting.†

Characteristics, n (%)	Before sIPTW				After sIPTW‡			
	MDRT group (n = 314)	non-MDRT group (n = 759)	P value	SMD	MDRT group (n = 307)	non-MDRT group (n = 755)	P value	SMD
**Gender, n (%)**								
Male	246 (78.3)	596 (78.5)	0.948	0.004	240 (78.1)	588 (78.0)	0.965	0.003
Female	68 (21.7)	163 (21.5)			67 (21.9)	166 (22.0)		
**Age, years**	44 (36, 54)	46 (37, 54)	0.236	0.099	46 (36, 56)	46 (37, 54)	0.966	0.001
**ECOG PS, n (%)**								
0	277 (88.2)	648 (85.4)	0.219	0.084	264 (85.9)	648 (85.8)	0.994	0.001
1	37 (11.8)	111 (14.6)			43 (14.1)	107 (14.2)		
**Post-EBV DNA (copies/ml)**	0 (0, 0)	0 (0, 39)	0.016	0.058	0 (0, 6)	0 (0, 20)	0.724	0.021
**Post-CRP (mg/L)**	2.64 (1.25, 8.43)	4.20 (1.46, 10.36)	0.005	0.119	2.89 (1.28, 9.05)	3.86 (1.40, 9.76)	0.393	0.018
**Post-LDH (U/L)**	185.6 (164.2, 216.5)	188.1 (163.6, 224.8)	0.314	0.085	185.9 (166.2, 224.6)	187.3 (163.2, 224.1)	0.787	0.023
**Post-SII**	895.9 (519.3, 1564.9)	764.0 (463.5, 1297.8)	0.006	0.133	841.0 (479.3, 1521.5)	793.7 (476.6, 1346.8)	0.353	0.071
**Post-PNI**	48.3 (45.4, 51.3)	48.3 (45.0, 50.9)	0.416	0.024	48.3 (45.1, 51.4)	48.2 (45.0, 50.9)	0.542	0.005
**Liver metastasis, n (%)**								
Yes	87 (27.7)	281 (37.0)	0.003	0.200	106 (34.5)	261 (34.6)	0.995	0.002
No	227 (72.3)	478 (63.0)			201 (65.5)	493 (65.4)		
**Bone metastasis, n (%)**			< 0.001	0.547			0.777	0.021
Yes	186 (59.2)	250 (32.9)			126 (41.2)	302 (40.1)		
No	128 (40.8)	509 (67.1)			181 (58.9)	452 (59.9)		
**Lung metastasis, n (%)**								
Yes	73 (23.2)	360 (47.4)	< 0.001	0.523	116 (37.8)	307 (40.7)	0.502	0.054
No	241 (76.8)	399 (52.6)			191 (62.2)	448 (59.3)		
**Other metastasis, n (%)**								
Yes	62 (19.7)	277 (36.5)	< 0.001	0.379	98 (31.9)	242 (32.0)	0.975	0.003
No	252 (80.3)	482 (63.5)			209 (68.1)	513 (68.0)		
**No. of metastatic lesions, n (%)**								
≤ 5	157 (50.0)	194 (25.6)	< 0.001	0.521	101 (33.0)	240 (31.8)	0.714	0.026
> 5	157 (50.0)	565 (74.4)			206 (67.0)	514 (68.2)		
**Cycles of CIT, n (%)**								
< 6	140 (44.6)	252 (33.2)	< 0.001	0.235	114 (37.2)	273 (36.2)	0.780	0.021
≥ 6	174 (55.4)	507 (66.8)			193 (62.8)	481 (63.8)		
**Metastatic lesion response, n (%)**								
CR	17 (5.4)	26 (3.4)	0.026	0.178	16 (5.3)	34 (4.5)	0.864	0.041
PR	192 (61.1)	526 (69.3)			206 (67.0)	505 (66.9)		
SD	105 (33.4)	207 (27.3)			85 (27.7)	216 (28.6)		

Abbreviation: sIPTW, stabilized inverse probability of treatment weighting; SMD, standardized mean difference; Post-, post-chemoimmunotherapy; ECOG PS, Eastern Cooperative Oncology Group performance status; EBV, Epstein-Barr virus; LDH, lactate dehydrogenase; CRP, C-reactive protein; SII, systemic immune-inflammation index; PNI, prognostic nutritional index; CR, complete response; PR, partial response; SD, stable disease; CIT, chemoimmunotherapy; MDRT, metastasis-directed radiotherapy.

†Percentages have been rounded and may not sum to 100%.

‡The total number of patients in the two groups varies slightly in the post-sIPTW pseudo-data set due to weight truncation.

Table S2. Details of first-line chemoimmunotherapy.

Treatment	MDRT group (n = 315)	non-MDRT group (n = 768)
Immunotherapy regimens, n (%)		
Camrelizumab	72 (22.9)	176 (22.9)
Toripalimab	131 (41.6)	273 (35.5)
Tislelizumab	70 (22.2)	174 (22.7)
Pembrolizumab	8 (2.5)	26 (3.4)
Penpulimab	2 (0.6)	15 (2.0)
Sintilimab	27 (8.6)	56 (7.3)
Nivolumab	3 (1.0)	15 (2.0)
Other	2 (0.6)	33 (4.3)
Chemotherapy regimens, n (%)		
GP	179 (56.8)	531 (69.1)
TP	93 (29.5)	163 (21.2)
PF	12 (3.8)	16 (2.1)
TPF	0	4 (0.5)
TPC	31 (9.8)	54 (7.0)

Abbreviations: MDRT, metastasis-directed radiotherapy; GP, gemcitabine and platinum; TP, docetaxel/paclitaxel and platinum; PF, platinum and 5-fluorouracil; TPF, docetaxel/paclitaxel, platinum, and 5-fluorouracil; TPC, docetaxel/paclitaxel, platinum, and capecitabine.

Table S3. Details of metastasis-directed radiotherapy.

Parameters	MDRT group (n = 315)
Radiotherapy modality, n (%)	
SBRT	191 (60.6)
IMRT	124 (39.4)
Irradiated metastatic sites, n (%)†	
Bone	169 (53.7)
Liver	68 (21.6)
Lung	53 (16.8)
Other	39 (12.4)
Extent of target coverage	
Comprehensive coverage (all residual active lesions targeted)	219 (69.5)
Partial coverage (cytoreductive irradiation of selected lesions)	96 (30.5)
Radiotherapy completion rate (%)	100
EQD2 of radiotherapy, median (IQR)	52 Gy (41–60 Gy)

†Percentages may sum to >100% as some patients received irradiation to multiple organ sites.

Abbreviations: MDRT, metastasis-directed radiotherapy; IMRT, intensity-modulated radiation therapy; SBRT, stereotactic body radiation therapy; EQD2, equivalent dose in 2Gy fraction; IQR, interquartile range.

Table S4. Univariable and multivariable analysis of progression-free survival assessing the impact of local and systemic treatment variations.

Variable	PFS			
	Univariable analysis		Multivariable analysis	
	HR (95 % CI)	P	HR (95 % CI)	P
**Local Treatment**				
MDRT	0.55 (0.45 ~ 0.66)	<0.001	0.55 (0.45~ 0.67)	<0.001
non-MDRT				
**Immunotherapy regimens**				
Pembrolizumab				
Toripalimab	1.36 (0.82 ~ 2.26)	0.232		
Tislelizumab	1.34 (0.80 ~ 2.25)	0.267		
Camrelizumab	1.35 (0.80 ~ 2.26)	0.258		
Penpulimab	1.26 (0.57 ~ 2.78)	0.562		
Sintilimab	1.28 (0.73 ~ 2.25)	0.385		
Nivolumab	1.75 (0.81 ~ 3.78)	0.152		
Other	1.72 (0.91 ~ 3.26)	0.095		
**Chemotherapy regimens**				
GP				
TP	0.93 (0.77 ~ 1.12)	0.441	1.00 (0.82 ~ 1.20)	0.961
PF	0.98 (0.60 ~ 1.59)	0.937	1.09 (0.67 ~ 1.76)	0.742
TPF	1.10 (0.27 ~ 4.42)	0.893	0.96 (0.24 ~ 3.87)	0.958
TPC	0.67 (0.47 ~ 0.95)	0.025	0.72 (0.50 ~ 1.03)	0.069

Abbreviations: MDRT, metastasis-directed radiotherapy; GP, gemcitabine and platinum; TP, docetaxel/paclitaxel and platinum; PF, platinum and 5-fluorouracil; TPF, docetaxel/paclitaxel, platinum, and 5-fluorouracil; TPC, docetaxel/paclitaxel, platinum, and capecitabine.

Table S5. Patterns and sIPTW-weighted balance of post-chemoimmunotherapy maintenance strategies.[†]

Maintenance Strategy	Before sIPTW			After sIPTW[‡]		
	non-MDRT group	MDRT group	SMD	non-MDRT group	MDRT group	SMD
IO-based regimens	563 (74.2%)	249 (79.3%)	0.121	555 (73.5%)	237 (77.3%)	0.087
Non-IO regimens	91 (12.0%)	31 (9.9%)	0.068	91 (12.1%)	29 (9.4%)	0.090
Observation	105 (13.8%)	34 (10.8%)	0.092	109 (14.4%)	41 (13.4%)	0.028

IO-based regimens include any treatment containing immune checkpoint inhibitors, either as monotherapy or combined with oral chemotherapy or targeted agents. Non-IO regimens refer to systemic regimens without immunotherapy (e.g., oral chemotherapy, targeted therapy, or their combination).

Abbreviation: IO, immunotherapy; sIPTW, stabilized inverse probability of treatment weighting; MDRT, metastasis-directed radiotherapy; SMD, standardized mean difference.

[†]Percentages have been rounded and may not sum to 100%.

[‡]The total number of patients in the two groups varies slightly in the post-sIPTW pseudo-data set due to weight truncation.

Table S6. E-value Sensitivity Analyses for Unmeasured Confounding on Survival Outcomes within the Target Trial Emulation Framework.

Survival Outcome	Observed Causal Estimate (95% CI)	E-value
PFS	0.64 (0.52 - 0.77)	2.08
OS	0.64 (0.45 - 0.86)	2.10

Abbreviations: PFS, progression-free survival; OS, overall survival.

Table S7. Univariable and multivariable analysis of progression-free survival among patients receiving consolidative MDRT.

Variable	Univariable analysis		Multivariable analysis	
	HR (95 % CI)	P	HR (95 % CI)	P
**Age**	0.99 (0.98 ~ 1.01)	0.459		
**ECOG PS**				
0				
1	1.20 (0.70 ~ 2.07)	0.501		
**Radiotherapy modality**				
IMRT				
SBRT	0.98 (0.69 ~ 1.39)	0.893		
**Metastatic burden**				
Oligometastatic				
Polymetastatic	1.99 (1.40 ~ 2.84)	<0.001	2.05 (1.43 ~ 2.92)	<0.001
**Tumor response**				
CR				
PR	2.11 (0.66 ~ 6.69)	0.206		
SD	3.09 (0.97 ~ 9.89)	0.057		
**Liver metastasis**				
No				
Yes	1.19 (0.80 ~ 1.76)	0.390		
**Lung metastasis**				
No				
Yes	0.86 (0.57 ~ 1.31)	0.479		
**Bone metastasis**				
No				
Yes	1.46 (1.01 ~ 2.12)	0.047	1.53 (1.05 ~ 2.22)	0.026
**Other metastasis**				
No				
Yes	1.25 (0.82~ 1.90)	0.298		

Abbreviation: IMRT, intensity-modulated radiation therapy; SBRT, stereotactic body radiation therapy; ECOG PS, Eastern Cooperative Oncology Group performance status; CR, complete response; PR, partial response; SD, stable disease.

Table S8. Hyper-parameters for the eight machine learning algorithms.

Algorithm	Optimized Parameters
LASSO Cox regression	'alpha' = 1, 'lambda' = 'lambda.min'
RSF	'mtry' = 2, 'nodesize' = 65, 'ntree' = 500
XGBoost	'max_depth' = 4, 'eta' = 0.01, 'nrounds' = 269, 'subsample' = 0.8
GBM	'n.trees' = 78, 'interaction.depth' = 3, 'shrinkage' = 0.05
CoxBoost	'stepno' = 35
Survivalsvm	'gamma.mu' = 0.10, 'kernel' = 'lin_kernel', 'margin' = 0.05
SuperPC	'threshold' = 0.5, 'n.components' = 1
plsRcox	'nt' = 3

The hyperparameters were automatically optimized to mitigate overfitting. Parameters not specified in the table are set to their default values in their respective packages.

Abbreviations: RSF, Random Survival Forest; XGBoost, Extreme Gradient Boosting; GBM, Gradient Boosting Machine; CoxBoost, cox model with likelihood-based boosting; Survivalsvm, Survival Support Vector Machine; SuperPC, Super Principal Components; plsRcox, Partial Least Squares Cox regression.

Table S9. Multicollinearity analysis of the final selected features in the machine learning models.

Multicollinearity metrics	Derivation cohort	External validation cohort
**Variance Inflation Factor**		
MDRT	1.08	1.10
Pre-EBV DNA	1.26	1.53
Post-EBV DNA	1.42	1.70
Pre-LDH	1.18	1.19
Post-CRP	1.10	1.17
No. of metastatic lesions	1.17	1.17
Metastatic lesion response	1.13	1.09
**Pairwise Spearman Correlations**		
Post-EBV DNA vs. Post-CRP	0.223	0.236
Post-EBV DNA vs. Pre-LDH	0.239	0.214
Pre-LDH vs. Post-CRP	0.211	0.162

Abbreviation: MDRT, metastasis-directed radiotherapy; EBV, Epstein-Barr virus; LDH, lactate dehydrogenase; CRP, C-reactive protein.

Table S10. Stratification of counterfactual baseline risk driven by recursive partitioning analysis.

Risk group	Patients, n (%)	Baseline risk score interval (log relative hazard)	Median baseline risk score (IQR)	3-year expected PFS without MDRT, % (95% CI)
Low-risk	185 (19.9%)	< -1.85	-2.164 (-2.458, -1.984)	72.5 (71.7, 73.4)
Intermediate-risk	564 (60.6%)	[-1.850, 0.057)	-1.039 (-1.432, -0.560)	35.1 (33.6, 36.5)
High-risk	182 (19.5%)	≥ 0.057	0.509 (0.277, 0.783)	1.5 (1.3, 1.7)

Table S11. Sensitivity analysis of baseline risk stratification consistency after omitting censoring weights.

Measure	Derivation cohort	External cohort
Spearman rank correlation between weighted and unweighted scores	0.99	0.99
Patients assigned to the same risk tier, %	92.4	92.1

Table S12. Mean estimated absolute risk reduction (ARR) with MDRT by risk tier and scoring approach.

Cohort	Risk tier	N, original	1-year ARR, %, original	2-year ARR, %, original	3-year ARR, %, original	N, unweighted	1-year ARR, %, unweighted	2-year ARR, %, unweighted	3-year ARR, %, unweighted
Derivation	Low-risk	185	7.0	11.0	15.8	170	7.1	11.6	16.5
Derivation	Intermediate-risk	564	14.2	15.9	20.9	551	14.5	16.4	21.6
Derivation	High-risk	182	8.4	4.9	4.9	210	7.8	4.1	4.4
External	Low-risk	25	6.5	10.3	14.7	24	6.3	10.1	14.5
External	Intermediate-risk	85	14.6	16.7	21.6	77	15.2	18.1	23.4
External	High-risk	42	6.1	5.3	5.7	51	6.6	5.2	5.9

The unweighted baseline prognostic score was refitted with the same candidate variables, hyperparameters, and tuning procedure as the primary score, omitting only the inverse probability of censoring weights. Risk tiers were re-derived by recursive partitioning analysis using the same settings as the primary analysis.

Table S13. Estimated overall and conditional treatment effects of MDRT on 3-year progression-free survival with robustness and sensitivity analyses.

Target population/RPA risk group	Estimated treatment effect, % (95% CI)	Effect with random perturbation, % (95% CI)	Robustness (CCC)	Sensitivity (E-value)	NNT
Overall cohort (ATE)	16.1% (8.9%, 26.2%)	15.9% (8.9%, 26.0%)	0.962	1.93	6.2
Low-risk (CATE)	14.7% (6.6%, 20.9%)	14.6% (6.9%, 21.1%)	0.958	4.2	6.8
Intermediate-risk (CATE)	21.6% (12.2%, 35.3%)	21.5% (12.0%, 35.9%)	0.944	2.3	4.6
High-risk (CATE)	5.7% (0.1%, 16.5%)	5.2% (0.3%, 15.8%)	0.990	1.4	17.6

Abbreviations: MDRT, metastasis-directed radiotherapy; ATE, average treatment effect; CCC, concordance correlation coefficient; CI, confidence interval; NNT, number needed to treat.

Table S14. Features used for machine learning algorithms development.

**All Features**	
Continuous features	Age (years); Pre-EBV DNA copy number (copies/ml); Pre-LDH (U/L); Pre-CRP levels (mg/L); Pre-NLR; Pre-PLR; Pre-SII; Pre-PNI; Post-EBV DNA copy number (copies/ml); Post-LDH (U/L); Post-CRP levels (mg/L); Post-NLR; Post-PLR; Post-SII; Post-PNI
Categorical features	Gender (men versus women); ECOG PS (0, 1); History of smoking (Yes versus No); History of alcohol abuse (Yes versus No); Liver metastasis (Yes versus No); Bone metastasis (Yes versus No); Lung metastasis (Yes versus No); Other metastasis (Yes versus No); No. of metastatic sites (≤ 2 versus > 2); No. of metastatic lesions (≤ 5 versus > 5); Cycles of CIT (< 6 versus ≥ 6); Metastatic lesion response after CIT (CR, PR, SD); MDRT (Yes versus No)
**Features in GBM**	
Continuous features	Pre-EBV DNA copy number (copies/ml); Pre-LDH (U/L); Post-EBV DNA copy number (copies/ml); Post-CRP levels (mg/L)
Categorical features	No. of metastatic lesions (≤ 5 versus > 5); Metastatic lesion response after CIT (CR, PR, SD); MDRT (Yes versus No)

Abbreviation: Pre-, pre-chemoimmunotherapy; Post-, post-chemoimmunotherapy; ECOG PS, Eastern Cooperative Oncology Group performance status; EBV, Epstein-Barr virus; LDH, lactate dehydrogenase; CRP, C-reactive protein; NLR, neutrophil-to-lymphocyte ratio; PLR, platelet-to-lymphocyte ratio; SII, systemic immune-inflammation index; PNI, prognostic nutritional index; CR, complete response; PR, partial response; SD, stable disease; CIT, chemoimmunotherapy; MDRT, metastasis-directed radiotherapy.

Table S15. Summary of the protocol for the hypothetical randomized trial and target trial emulation (Population-level survival analysis).

Component	Hypothetical randomized trial	Target Trial Emulation
**Eligibility**	**Subjects must meet all of the following conditions to be selected:**<br>(1) Pathologically confirmed nasopharyngeal carcinoma (NPC) with a documented history of receiving prior definitive treatment for the primary tumor;<br>(2) Presence of exclusively distant metastasis, defined as distant metastasis without synchronous locoregional recurrence (no recurrence at the primary site or regional lymph nodes) at the time of metastasis diagnosis, strictly confirmed via pathological biopsy or comprehensive imaging evaluation (e.g., PET-CT, MRI);<br>(3) Receipt of at least 2 cycles of standard first-line chemoimmunotherapy (CIT);<br>(4) Age ≥18 years;<br>(5) Eastern Cooperative Oncology Group (ECOG) performance status of 0–1;<br>(6) Expected life expectancy ≥ 4 months;<br>(7) Adequate blood, liver and kidney function.<br>**Subjects who meet one of the following conditions will be excluded for this study:**<br>(1) Presence of local recurrence at the primary site or regional lymph node metastasis at the time of distant metastasis diagnosis;<br>(2) Progressive disease (PD) prior to or exactly at the post-CIT efficacy evaluation;<br>(3) History of prior or synchronous malignancies;<br>(4) Receipt of any metastasis-directed radiotherapy (MDRT) prior to the post-CIT evaluation.	**Inclusion criteria:**<br>(1) Pathologically confirmed nasopharyngeal carcinoma (NPC) with a documented history of receiving prior definitive treatment for the primary tumor;<br>(2) Evidence of exclusively distant metastasis, defined as distant metastasis without synchronous locoregional recurrence (no recurrence at the primary site or regional lymph nodes) at the time of metastasis diagnosis, based on a comprehensive evaluation via pathology or imaging;<br>(3) Received at least two cycles of first-line chemoimmunotherapy (CIT);<br>(4) Age ≥18 years;<br>(5) Eastern Cooperative Oncology Group (ECOG) performance status of 0–1.<br>**Exclusion criteria:**<br>(1) Local recurrence at the primary site or regional lymph node metastasis at the time of distant metastasis diagnosis;<br>(2) Progressive disease (PD) at any time during first-line CIT or at the initial post-CIT efficacy evaluation;<br>(3) Incomplete medical records or follow-up documentation;<br>(4) Combined with other malignancies;<br>(5) Receipt of any metastasis-directed radiotherapy (MDRT) prior to the post-CIT evaluation.
**Time zero**	The date of randomization, which occurs at the	The date of the post-CIT efficacy

	post-CIT efficacy evaluation juncture after confirmation of non-progressive disease.	evaluation, defined as the first efficacy assessment following completion or termination of first-line CIT, at which non-progression was confirmed per RECIST 1.1.
**Treatment strategies**	**Study group:** CIT plus consolidative MDRT, initiated within 1.5 months after randomization;<br>**Control group:** CIT alone, with no MDRT initiated within 1.5 months after randomization.	**Study group:** initiation of MDRT within the 1.5-month grace period after T0;<br>**Control group:** CIT alone, with no initiation of MDRT within the 1.5-month grace period after T0; subsequent treatment was unrestricted.
**Grace period**	A 1.5-month window after randomization during which MDRT must be initiated, mirroring the time required for radiotherapy preparation in routine practice.	A 1.5-month window after T0, prespecified on clinical grounds. Simulation, target delineation, treatment planning, and quality assurance before MDRT delivery routinely require approximately 2 to 6 weeks. Patients who experienced progression or death within the window were retained in both strategy arms and contributed events.
**Treatment assignment**	Eligible subjects are randomized in a 1:3 (MDRT to non-MDRT) ratio to receive either CIT plus MDRT or CIT alone at the post-CIT evaluation juncture.<br>The stratification factors for randomization include:<br>1. Objective tumor response to CIT (CR/PR vs. SD);<br>2. Metastatic tumor burden (e.g., oligometastatic vs. polymetastatic);<br>3. EBV DNA status (undetectable vs. detectable).	Patients are classified on the basis of their observed treatment strategies during the 1.5-month grace period. Patients were cloned and assigned to each treatment arm to emulate randomization. After censoring patients who did not adhere to their assigned treatments during the grace period and applying inverse probability of censoring weighting (IPCW), it was assumed that patients in each treatment arm were comparable.
**Blinding**	Participants and treating physicians are aware of the strategy they are assigned to	Same as hypothetical trial.
**Follow-up**	Follow-up begins precisely at randomization (the post-CIT evaluation juncture). Patients are followed up with standard clinical and imaging assessments (every 3 months).	Follow-up begins exactly at Time Zero (T0), strictly defined as the date of the post-CIT efficacy evaluation.<br>The follow-up ends at the earliest of:

	The follow-up ends at the earliest of: death, loss to follow-up, or administrative end of the trial.	death, loss to follow-up, or administrative end of the observational follow-up.
**Outcome**	**Primary endpoint:**<br>Progression-free survival (PFS), defined as the time from randomization to the first occurrence of disease progression according to RECIST 1.1 or death from any cause (whichever occurs first).<br>**Secondary endpoint:**<br>Overall survival (OS), defined as the time from randomization to death from any cause.	**Primary endpoint:**<br>PFS, defined as the interval from T0 to the first documented disease progression per RECIST 1.1 or death from any cause, whichever occurred first; patients without an event were censored at the last assessment or at the administrative end of follow-up.<br>**Secondary outcome:**<br>OS, defined as the interval from T0 to death from any cause; surviving patients were censored at the last follow-up or at the administrative end of follow-up.
**Censoring rules**	Administrative censoring at the end of the trial or loss to follow-up.	Administrative censoring at the end of observational follow-up or loss to follow-up, plus artificial censoring upon deviation from the assigned strategy as defined under Treatment assignment. Artificially censored clones contributed follow-up until deviation and were accounted for by inverse probability of censoring weighting.
**Causal contrast**	**Intention-to-treat (ITT) effect for efficacy:**<br>The average treatment effect of being randomly assigned to either CIT plus consolidative MDRT or CIT alone, regardless of actual adherence to the assigned treatment strategy.	The average treatment effect of initiating the assigned strategy (CIT plus MDRT vs. CIT alone) within the grace period, regardless of subsequent adherence. Initiation of MDRT after the grace period does not count as the MDRT strategy. This is achieved via the cloning-censoring-weighting approach, which is akin to an ITT analysis and preserves the intention-to-treat principle.
**Assumptions**	**Consistency, exchangeability, and positivity** assumptions are inherently satisfied through the prospective randomization process and strictly controlled study design.	**Consistency**: An individual's observed survival outcome under their actual treatment strategy equals their counterfactual potential outcome,

		predicated on well-defined interventions.<br>**Exchangeability** (No unmeasured confounding): Assumes all critical prognostic confounders are captured and effectively balanced via IPCW. The robustness of this assumption against potential unmeasured confounding was quantitatively validated using E-values.<br>**Positivity**: Assumes every eligible patient at T0 has a non-zero probability of receiving either strategy. Practical positivity was enforced by symmetrically truncating unstabilized IPCW values at the 1st and 99th percentiles.<br>**Model specification**: Relies on the correct parametric specification of the models utilized to estimate the IPCW weights.
**Sample size**	The sample size calculation adheres to the ideal target trial framework. Based on empirical baseline parameters and literature review, the median progression-free survival (PFS) was assumed to be 23.0 months for patients receiving CIT plus MDRT, and 16.0 months for patients receiving CIT alone. Assuming an allocation ratio of 1:3, a 24-month accrual and an additional 36-month follow-up with a 5% annual loss to follow-up, the estimated sample size for this target trial is 443 patients (111 in the MDRT group and 332 in the non-MDRT group) [4, 5]. This sample size would target 348 progression events to provide 80% power to detect a hazard ratio (HR) for PFS of 0.70 at a two-sided significance level of 0.05. The sample size estimation was conducted using PASS 15.0.5 software.	The sample size for the target trial emulation intentionally exceeded the minimum theoretical requirement of the ideal target trial. All available eligible patients were included to maximize statistical power, enhance the robustness of causal estimates, and improve broad generalizability. The final emulation base cohort comprised 1083 patients, with 315 patients allocated to the MDRT group (264 from Center 1 and 51 from Center 2) and 768 patients in the non-MDRT group (667 from Center 1 and 101 from Center 2).
**Multiplicity**	To strongly control the overall family-wise error rate (FWER) across the primary and secondary endpoints, a strict hierarchical	Same as hypothetical trial.

	(fixed-sequence) testing procedure is applied. Specifically, the primary endpoint (PFS) is tested first at a two-sided significance level of 0.05. The secondary endpoint (OS) is subsequently evaluated at the 0.05 level only if the PFS benefit achieves statistical significance. If the PFS result is not statistically significant, formal hypothesis testing terminates, and any subsequent evaluation of OS is considered strictly exploratory.<br>No interim analysis is considered or planned.	
**Statistical analysis**	The hypothesis testing for the time-to-event distributions (PFS and OS) is evaluated in the intention-to-treat (ITT) population using a stratified log-rank test (adjusting for the prespecified randomization stratification factors). The hazard ratio (HR) and corresponding 95% confidence intervals (CI) are estimated using a stratified Cox proportional hazards model. Survival curves for each treatment group are estimated using the standard Kaplan-Meier method. A two-sided P value ≤ 0.05 indicates statistical significance, strictly governed by the aforementioned hierarchical testing procedure.	Given that the cloning step and the enforced identical treatment assignment during the 1.5-month grace period inherently violate the proportional hazards assumption, restricted mean survival time (RMST) is utilized as the primary metric to assess robust differences in PFS and OS. HRs derived from IPCW-weighted Cox proportional hazards models are additionally calculated for reference. To rigorously account for the artificially inflated sample size and deflated variance generated by the cloning procedure, 95% CIs and P values are computed utilizing non-parametric bootstrapping with 1,000 resamples. Multiple sensitivity analyses are conducted to verify the robustness of the primary estimates, including a 1.5-month conditional landmark analysis using stabilized inverse probability of treatment weighting (sIPTW), propensity score matching (PSM), and E-value quantification for unmeasured confounding.

Table S16. Specification and distribution parameters of the inverse probability of censoring weighting in the target trial emulation.

Item	Non-MDRT strategy arm	MDRT strategy arm
Artificial censoring definition	Initiation of MDRT during the 1.5-month grace period	Failure to initiate MDRT by the end of the 1.5-month grace period
Clones assigned, n	1083	1083
Clones artificially censored, n (%)	296 (27.3)	778 (71.8)
Clones remaining after the grace period, n (%)	787 (72.7)	305 (28.2)
Censoring (denominator) model	Arm-specific logistic regression	Arm-specific logistic regression
Covariates in the censoring models	ECOG performance status, gender, age, liver metastasis, bone metastasis, lung metastasis, other metastasis, number of metastatic lesions, metastatic lesion response to CIT, cycles of CIT, post-EBV DNA, post-CRP, post-LDH, post-SII, and post-PNI	Same covariate set as the non-MDRT arm
Weight form	Unstabilized: 1 / P (adherence | covariates)	Unstabilized: 1 / P (adherence | covariates)
Truncation thresholds (1st / 99th percentile)	1.051 / 3.392	1.435 / 15.945
Pre-truncation weights, mean (SD)	1.487 (0.511)	5.036 (3.280)
Pre-truncation weights, median (IQR)	1.312 (1.158–1.566)	3.951 (2.701–6.614)
Pre-truncation weights, min–max	1.030–4.651	1.312–24.613
Post-truncation weights, mean (SD)	1.482 (0.488)	5.001 (3.122)
Post-truncation weights, median (IQR)	1.312 (1.158–1.566)	3.951 (2.701–6.614)
Post-truncation weights, min–max	1.051–3.392	1.435–15.945
Clones truncated, %	2.0	2.0
Weighted pseudo-population size[‡]	1080.6	1088.7
Effective sample size among adherent clones, n/N (efficiency)[§]	732.9/787 (93.1%)	218.5/305 (71.7%)

[‡] Sum of post-truncation weights among adherent clones; the target value is the original cohort size (n = 1083). [§] Effective sample size = $(\Sigma w)^2 / \Sigma w^2$, calculated with post-truncation weights among adherent clones.

Abbreviations: MDRT, metastasis-directed radiotherapy; EBV, Epstein-Barr virus; LDH, lactate dehydrogenase; CRP, C-reactive protein; SII, systemic immune-inflammation index; PNI, prognostic nutritional index; SD, standard deviation; IQR, interquartile range.

Table S17. Sample sizes and hyperparameter specifications of the arm-specific gradient boosting machine models within the T-learner framework (derivation cohort).

Characteristic	MDRT arm (mu1)	No-MDRT arm (mu0)
Clones in training set, n	931	931
Events (unweighted), n	98	431
Events (IPCW-weighted), n	227.9	453.5
Effective sample size after IPCW	444.1	858.5
Interaction depth	2	2
Minimum observations per terminal node	30	30
Shrinkage	0.05	0.05
Boosting iterations (arm-specific 5-fold CV)	168	132

Both arm-specific models were trained on the full set of clones assigned to the corresponding strategy within the emulated trial, with arm-specific inverse probability of censoring weights (IPCW) applied and truncated at the 1st and 99th percentiles. The effective sample size was computed as (sum of weights)^2 / sum of squared weights.

References

1. Cupp MA, Cariolou M, Tzoulaki I, Aune D, Evangelou E, Berlanga-Taylor AJ. Neutrophil to lymphocyte ratio and cancer prognosis: an umbrella review of systematic reviews and meta-analyses of observational studies. BMC Med. 2020;18(1):360.
2. Yang D, Li P, Meng Z, et al. Combined pretreatment neutrophil-lymphocyte ratio and platelet-lymphocyte ratio predicts survival and prognosis in patients with non-metastatic nasopharyngeal carcinoma: a retrospective study. Sci Rep. 2024;14(1):9898.
3. Guo J, Yang Q, Jiang Q, Gu LW, Lin HX, Guo L. Integrating Baseline Nutritional and Inflammatory Parameters with Post-Treatment EBV DNA Level to Predict Outcomes of Patients with De Novo Metastatic Nasopharyngeal Carcinoma Receiving Chemotherapy Combination PD-1 Inhibitor. Nutrients. 2023;15(19):4262.
4. He SQ, Lv SH, Wen SQ, et al. Efficacy of metastatic lesion radiotherapy in de novo metastatic nasopharyngeal carcinoma patients receiving local regional radiotherapy and chemo-immunotherapy: a multicenter retrospective study. Radiother Oncol. 2025;213:111160.
5. Zhang X, Yan J, Lei Q, et al. A Randomized, Multicenter, Phase 2 Trial of Camrelizumab With or Without Metastasis-directed Stereotactic Body Radiation Therapy in Recurrent or Metastatic Nasopharyngeal Carcinoma. Int J Radiat Oncol Biol Phys. 2025;121(4):980-990.
